# Cembrane Diterpenes Possessing Nonaromatic Oxacycles from the Hainan Soft Coral *Sarcophyton mililatensis*

**DOI:** 10.3390/ijms24031979

**Published:** 2023-01-19

**Authors:** Ling Zhang, Min Yang, Zi-Hui Chen, Zeng-Yue Ge, Song-Wei Li, Xian-Yun Yan, Li-Gong Yao, Lin-Fu Liang, Yue-Wei Guo

**Affiliations:** 1 College of Materials Science and Engineering, Central South University of Forestry and Technology, Changsha 410004, China; 2 State Key Laboratory of Drug Research, Shanghai Institute of Materia Medica, Chinese Academy of Sciences, 555 Zu Chong Zhi Road, Zhangjiang Hi-Tech Park, Shanghai 201203, China; 3 Shandong Laboratory of Yantai Drug Discovery, Bohai Rim Advanced Research Institute for Drug Discovery, Yantai 264117, China; 4 Open Studio for Druggability Research of Marine Natural Products, Pilot National Laboratory for Marine Science and Technology (Qingdao), 1 Wenhai Road, Aoshanwei, Jimo, Qingdao 266237, China; 5 Collaborative Innovation Center of Yangtze River Delta Region Green Pharmaceuticals and College of Pharmaceutical Science, Zhejiang University of Technology, Hangzhou 310014, China

**Keywords:** soft coral, *Sarcophyton mililatensis*, cembrane diterpene, absolute configuration, TNF-*α* inhibitory, binding mode

## Abstract

Documents on the chemical composition of the soft coral *Sarcophyton mililatensis* are sparse. The present investigation of the Hainan soft coral *S. mililatensis* resulted in the discovery of six new cembrane diterpenes, sarcoxacyclols A–F (**1**–**6**) and four known analogs (**7**–**10**). Their structures were elucidated by extensive spectroscopic analysis along with a comparison with the data in current literature. The nonaromatic oxacycles in their structures were the rarely found tetrahydrofuran ether across C-1 and C-12 and tetrahydropyran ether across C-1 and C-11, respectively. Moreover, the absolute configuration of compound **4** was established unambiguously by X-ray diffraction analysis using Ga Kα radiation (*λ* = 1.34139 Å). Based on the biogenetical consideration, the absolute configurations of other five new compounds were tentatively assumed. Assessment of the bioactivity for these secondary metabolites revealed that compound **1** exhibited significant tumor necrosis factor (TNF)-*α* inhibitory activity (IC_50_ = 9.5 μmol/L), similar to the positive control dexamethasone (IC_50_ = 8.7 μmol/L), but no obvious cytotoxicity towards RAW264.7 cells (CC_50_ > 50 μmol/L). The preliminary molecular docking suggested the crucial roles of the hydroxyl and acetoxyl groups in the computational prediction of the binding mode between the diterpene and the protein.

## 1. Introduction

Nonaromatic oxacycles such as dihydrofuran and oxepane are frequently encountered structural units in a number of pharmacologically active marine natural products [1]. For instance, the FDA-approved anti-cancer drug Yondelis is a eunicellane diterpene that possesses a dihydrofuran motif [2]. Cembrane diterpenes, which could be regarded as the precursors of eunicellane diterpenes [3], also possess nonaromatic oxacycles. The majority of nonaromatic oxacycles in cembranes usually appear as five- to seven-membered lactones, while the minority of remainder are tetrahydrofuran and tetrahydropyran ethers [4]. Different structural features of tetrahydrofuran and tetrahydropyran ethers could be attributed to the various linkages in the cembrane diterpenes [5], including bridging carbons C-1/C-11 [6,7,8], C-1/C-12 [7,8,9], C-2/C-12 [6], C-3/C-7 [10], C-3/C-14 [11], C-4/C-8 [12], C-4/C-15 [13,14,15], C-8/C-11 [14], C-10/C-14 [6], and C-12/C-15 [7]. Among them, the number of cembrane diterpenes possessing a tetrahydrofuran ether across C-1 and C-12 and a tetrahydropyran ether across C-1 and C-11 were ca. 20 [6,7,8,9], which were extremely rare as compared to the large quantity of cembrane family members (over 600) [4]. The PTP1B inhibitory [7], antitumor [6,7], antibacterial [7], and antifouling [6] potencies of the above-mentioned two groups of cembranes were assessed. As a result, compounds such as sinulariol Z and (2*E*,7*E*)-4,11-dihydroxy-1,12-oxidocembra-2,7-diene could be developed as potential candidates for nontoxic antifoulants [6].

The assignment of absolute configuration to highly flexible cembrane diterpenes containing diverse functional groups remain challenging. Usually, X-ray crystallography techniques [16] and chemical derivatization approaches using chiral agents [17] are the primary choices [4]. However, the scarcity of isolates hampered the use of the two methods mentioned above in some cases. Instead, chiroptical properties of cembrane diterpenes, including electronic circular dichroism (ECD) aided by quantum chemical calculations, provided alternative approaches to establish the absolute configurations of this type of natural macrocyclic compounds [18]. Among the various calculation methods, the time-dependent density functional theory (TDDFT) ECD calculation is a powerful tool that has been frequently used. Till now, the efficiency of TDDFT ECD calculation has been demonstrated in the numerous stereochemical studies of conformationally flexible cembrane diterpenes [19,20,21] and their dimers [22,23,24].

**Figure 1:**
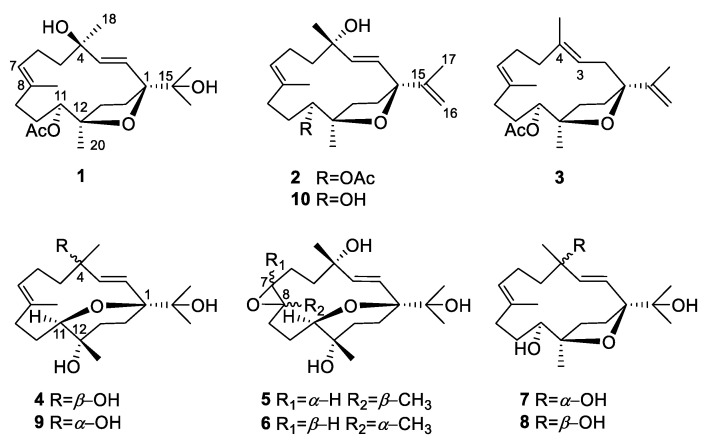
Structures of compounds **1**–**10**.

In the course of our ongoing research aiming for structurally novel and pharmacologically active secondary metabolites from South China Sea marine fauna [25], we encountered the soft coral *Sarcophyton mililatensis*, which was collected from Xigu Island, Hainan Province, China. As far as we know, there are only six reports focused on the chemical constituents of *S. mililatensis* [26,27,28,29,30,31], indicating documents on chemical composition of this species are sparse. Our previous chemical investigations on the title animals resulted in the discovery of an array of diterpenes with four carbon frameworks [26,27,28,29], involving an unprecedented tricyclo[11.3.0.0^2,16^]hexadecane carbon skeleton [28]. These studies disclosed the productivity of this species and inspired us to conduct further research on the title species, especially for structurally intriguing diterpenes. In the present study, ten cembrane diterpenes possessed different types of nonaromatic oxacycles. Among them, sarcoxacyclols A–F (**1**–**6**) were new compounds (Figure 1). As follows are the descriptions of the isolation, structural elucidation, and bioactivity screening of these secondary metabolites.

## 2. Results and Discussion

Freshly collected animals of *S. mililatensis* were immediately placed at –20 °C and kept frozen prior to extraction. The frozen samples were then extracted three times and the combined extract was separated by multiple rounds of chromatography to yield six new diterpenes, sarcoxacyclols A–F (**1**–**6**), along with four known related analogs **7**–**10** (Figure 1). These known compounds were readily identified as sarcophytrols M (**7**), P (**8**), Q (**9**) [7], and (2*E*,7*E*)-4,11-dihydroxy-l,12-oxidocembra-2,7,15,-triene (**10**) [9], respectively, by comparison of the NMR data and optical rotation with those reported in the literature.

**Figure 2:**
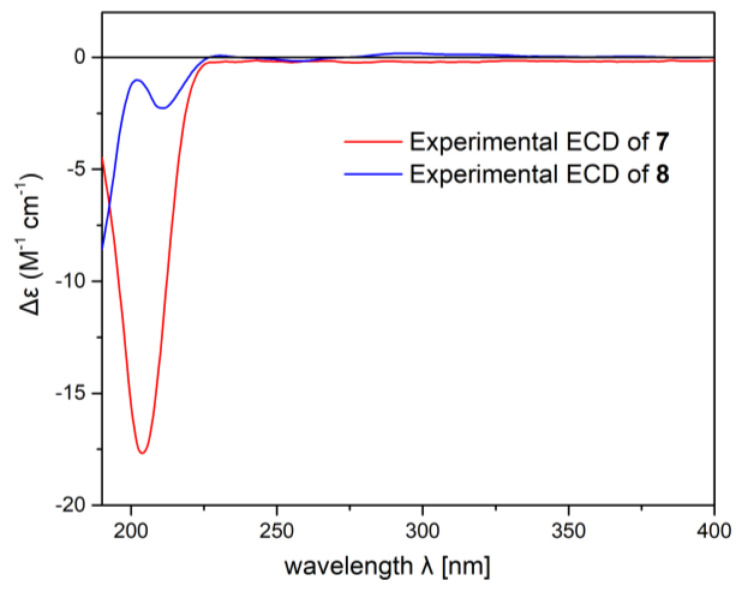
Experimental ECD spectra of compounds **7** (red) and **8** (blue) in MeCN.

In our previous work, the absolute configuration of sarcophytrol M (**7**), a cembrane diterpene possessing a furan ether isolated from the South China Sea soft coral *Sarcophyton trocheliophorum*, was determined as 1*S*,4*S*,11*S*,12*R* by the modified Mosher’s method [7]. Based on the C-4 epimeric relationship between sarcophytrols M (**7**) and P (**8**), the absolute configuration of compound **8** could be assigned as 1*S*,4*R*,11*S*,12*R*, which was further confirmed by the same Cotton effects at ca. 208 nm in their experimental ECD spectra (Figure 2) in the present study.

**Figure 3:**
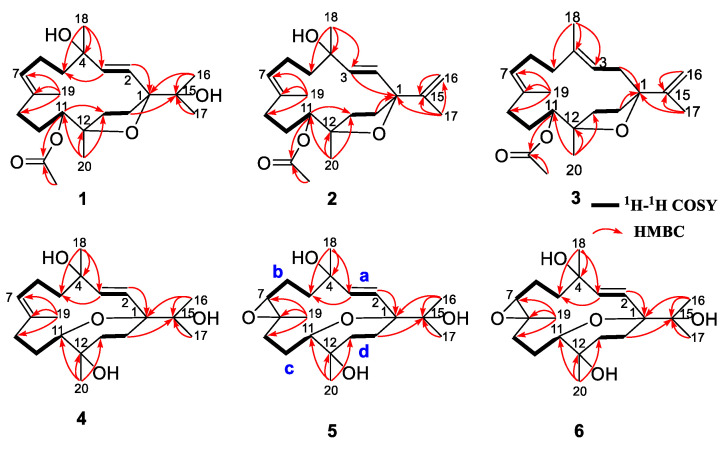
Selected key COSY and HMBC correlations for compounds **1**–**6**.

**Figure 4:**
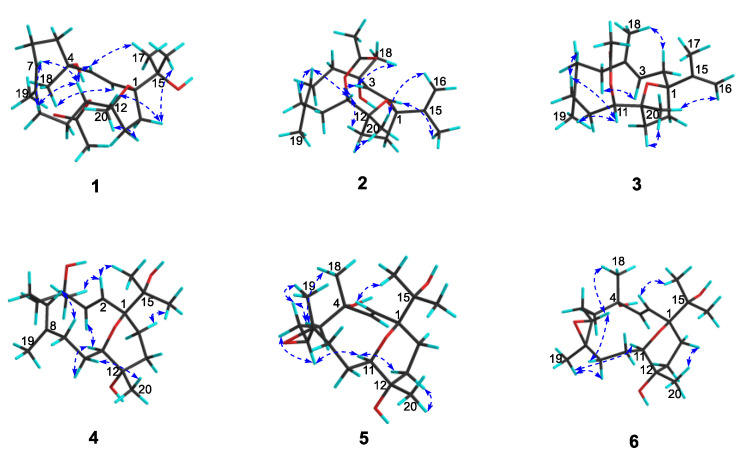
Selected key ROESY correlations for compounds **1**–**6**.

Sarcoxacyclol A (**1**), which was obtained as a colorless oil, possessed the molecular formula C_22_H_36_O_5_ on the basis of its HRESIMS ion peak at *m*/*z* 403.2448 ([M+Na]^+^, calcd. for C_22_H_36_O_4_Na, 403.2455), requiring five degrees of unsaturation. As revealed by the ^1^H and ^13^C NMR data (Table 1), there were one disubstituted double bond [*δ*_H_ 5.55 (1H, d, *J* = 15.8 Hz, H-2) and 6.11 (1H, d, *J* = 15.8 Hz, H-3); *δ*_C_ 129.8 (CH, C-2), 137.4 (CH, C-3)], one trisubstituted double bond [*δ*_H_ 5.42 (1H, dd, *J* = 4.3, 10.5 Hz, H-7); *δ*_C_ 130.1 (CH, C-7), 132.1 (qC, C-8)], and one acetyl group [*δ*_H_ 2.00 (3H, s, H_3_-OAc); *δ*_C_ 21.4 (CH_3_, *C*H_3_-OAc), 171.2 (qC, *C*O-OAc)] in the molecule of compound **1**. These functionalities accounted for three degrees of unsaturation. Thus, the remaining two degrees of unsaturation suggested the bicyclic framework of this compound. In fact, the NMR data of compound **1** closely resembled those of the co-occurring sarcophytrol P (**8**) [7], which was a cembrane diterpene that possessed a tetrahydrofuran ether across C-1 and C-12 from the South China Sea soft coral *S. trocheliophorum*, revealing that they were structural analogs. Careful analysis of their NMR data revealed the only difference was the presence of an acetoxyl group at C-11 in compound **1** instead of the hydroxyl in compound **8**. This replacement was supported by the diagnostic HMBC correlations of H-11 (*δ*_H_ 5.07), *C*O-OAc (*δ*_C_ 171.2), C-10 (*δ*_C_ 27.3), and C-12 (*δ*_C_ 84.5) (Figure 3). The observed NOE cross-peaks of H_3_-18 (*δ*_H_ 1.34)/H-2 (*δ*_H_ 5.55), H-2/H_3_-16 (*δ*_H_ 1.12), H_3_-16/H-14b (*δ*_H_ 2.31) and H-14b/H_3_-20 (*δ*_H_ 1.16) suggested the same orientation of H_3_-16, H_3_-18, and H_3_-20 (Figure 4). Whereas the lack of the NOE correlation of H-11 (*δ*_H_ 5.07)/H_3_-20 and the presence of H-3 (*δ*_H_ 6.11)/H-11 placed H-11 and H_3_-20 on the opposite faces of this molecule. Therefore, compound **1** was the 11-acetylation derivative of compound **8**, which was consistent with their 42 mass unit difference. Considering their structural relationship, the absolute configuration of compound **1** was tentatively supposed to be the same as that of compound **8** (Figure 1).

Sarcoxacyclol B (**2**) was isolated as a colorless oil and had a molecular formula of C_22_H_34_O_4_ as established by the pseudo-molecular ion peak at *m*/*z* 385.2361 ([M+Na]^+^, calcd. for C_22_H_34_O_4_Na, 385.2349) in its HRESIMS spectrum, requiring six degrees of unsaturation. The ^1^H and ^13^C NMR spectra (Table 1) of compound **2** showed resonances attributed to one disubstituted double bond [*δ*_H_ 5.68 (1H, d, *J* = 15.5 Hz, H-2), 5.92 (1H, d, *J* = 15.5 Hz, H-3); *δ*_C_ 130.4 (CH, C-2), 136.4 (CH, C-3)], one trisubstituted double bond [*δ*_H_ 5.37 (1H, dd, *J* = 6.2, 8.8 Hz, H-7); *δ*_C_ 128.3 (CH, C-7), 132.6 (qC, C-8)], one terminal double bond [*δ*_H_ 4.71 (1H, s, H-16a), 4.96 (1H, s, 16b); *δ*_C_ 109.4 (CH_2_, C-16), 149.5 (qC, C-15)], one acetyl [*δ*_H_ 2.05 (3H, s, H_3_-OAc); *δ*_C_ 21.4 (CH_3_, *C*H_3_-OAc), 171.2 (qC, *C*O-OAc)]. All the above-mentioned functionalities accounted for four degrees of unsaturation; the left two indicated that compound **2** was a bicyclic diterpene. Indeed, the NMR data of compound **2** were almost superimposed on those of compound **1**, suggesting the structural similarity of both compounds. Further comparison of their NMR data revealed that their gross structures differed at the substituent attached to C-1, which was a propen-2-yl group in compound **2** whereas a peculiar isopropyl alcohol moiety in compound **1**. This propen-2-yl group was assigned to C-1 based on the HMBC correlations from H_3_-17 (*δ*_H_ 1.71) to C-1 (*δ*_C_ 88.1), C-15 (*δ*_C_ 149.5) and C-16 (*δ*_C_ 109.4) (Figure 3). Thus, the planar structure of compound **2** was determined as the dehydration derivative of compound **1**. The *cis*-orientation of the propen-2-yl group and CH_3_-20 was deduced from the characteristic NOE cross-peak of H-16b (*δ*_H_ 4.96)/H_3_-20 (*δ*_H_ 1.18). The strong NOE correlations between H-2 (*δ*_H_ 5.68) and H_3_-17 (*δ*_H_ 1.71) and between H-3 (*δ*_H_ 5.92) H_3_-18 (*δ*_H_ 1.28) implied that H_3_-17 and H_3_-18 were *trans*-oriented. These NOE correlations (Figure 4) indicated that compound **2** and the co-isolated diterpene sarcophytrol M (**7**) [7] shared the same configurations for the four chiral centers C-1, C-4, C-11, and C-12. Tentatively, the absolute configuration of compound **2** was assumed to be 1*S*,4*S*,11*S*,12*R*.

Sarcoxacyclol C (**3**) was purified as a colorless oil with a molecular formula of C_22_H_34_O_3_, which was inferred from the HREIMS data (*m*/*z* 346.2507, M^+^, calcd. for C_22_H_34_O_3_, 346.2508). Its ^1^H NMR data (Table 1) revealed the presence of three vinyl methyls at *δ*_H_ 1.63 (3H, s, H_3_-19), 1.56 (3H, s, H_3_-18), and 1.75 (3H, s, H_3_-17), one methyl linked to a tertiary carbon at *δ*_H_ 1.18 (3H, s, H_3_-20), one methyl of an acetyl group at *δ*_H_ 2.06 (3H, s, H_3_-OAc), one proton linked to an oxygenated carbon at *δ*_H_ 4.88 (1H, d, *J* = 10.2 Hz, H-11), and four olefinic protons at *δ*_H_ 4.80 (1H, s, 16a), 5.10 (1H, s, H-16b), 5.12 (1H, t, *J* = 6.0 Hz, H-3), and 5.18 (1H, t, *J* = 6.0 Hz, H-7), which were indicative of two trisubstituted double bonds and one terminal double bond. The ^13^C NMR spectrum displayed 22 signals consisting of five methyls (*δ*_C_ 16.8, 17.6, 20.1, 21.5, and 21.8), eight methylenes (including seven aliphatic *δ*_C_ 24.9, 27.5, 32.0, 33.8, 35.3, 35.7, and 38.0, and one olefinic *δ*_C_ 110.1), three methines (including one oxygenated *δ*_C_ 75.7, two olefinic *δ*_C_ 119.9, and 126.2), and six quaternary carbons (including two oxygenated *δ*_C_ 83.5, 89.6, three olefinic *δ*_C_ 132.9, 136.6, 150.2, and one carbonyl *δ*_C_ 171.3) (Table 1). These abovementioned structural features of compound **3** were reminiscent of the co-isolated compound **2**. According to the interpretation of the 2D NMR spectra (Figure 3 and Figure 4), compounds **3** and **2** differed by the double bond at C-2/C-3 in compound **2** shifting to C-3/C-4 in compound **3** accompanied with the disappearance of the hydroxyl at C-4. This alteration was supported by the consecutive spinning proton system from H_2_-2 (*δ*_H_ 2.14 m, 2.35 m) to H-3 (*δ*_H_ 5.12) along with the key HMBC correlations of H_2_-2/C-1 (*δ*_C_ 89.6), H_3_-18 (*δ*_H_ 1.56)/C-3 (*δ*_C_ 119.9), H_3_-18/C-4 (*δ*_C_ 136.6), and H_3_-18/C-5 (*δ*_C_ 38.0) (Figure 3). The NOESY spectra of diterpenes **2** and **3** (Figure 4) showed similar distributions of NOE cross-peaks, implying they shared the same configurations for the three chiral carbons C-1, C-11, and C-12. Based on the biogenetical consideration, the absolute configuration of compound **3** was tentatively supposed to be 1*S*,11*S*,12*R*, and its structure was shown as depicted in Figure 1.

**Figure 5:**
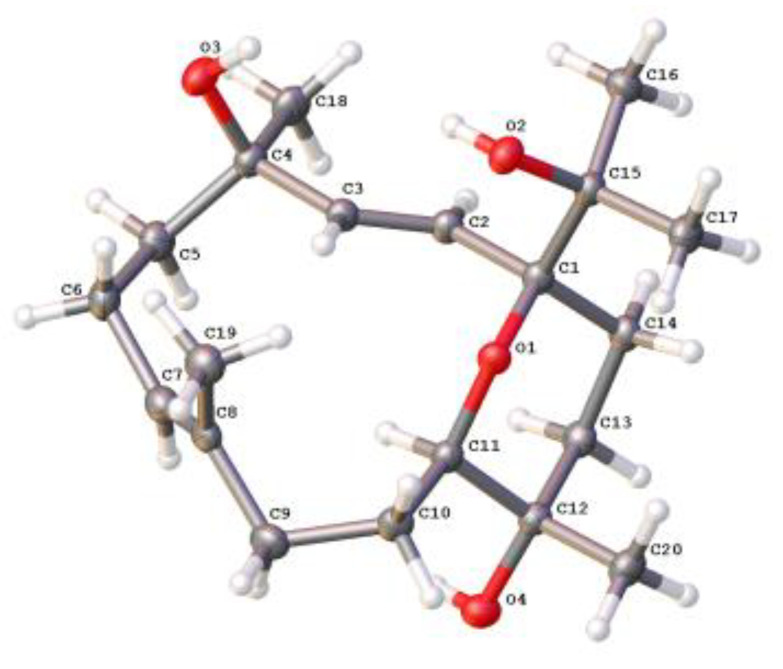
Perspective ORTEP drawings of X-ray structures of compound **4**.

Sarcoxacyclol D (**4**), colorless crystals, displayed a pseudo-molecular ion peak in the HRESIMS spectrum at *m*/*z* 361.2348 ([M+Na]^+^, calculated for C_20_H_34_O_4_Na, 361.2349), indicating that compound **4** possessed the same molecular formula C_20_H_34_O_4_ as that of the co-isolate sarcophytrol Q (**9**) [7]. Thus, four degrees of unsaturation were determined for this diterpene. The ^1^H and ^13^C NMR data (Table 2) revealed the presence of one trisubstituted double bond [*δ*_H_ 5.23 (1H, dd, *J* = 5.1, 10.2 Hz, H-7); *δ*_C_ 131.0 (CH, C-11), 132.6 (qC, C-8)], one disubstituted double bond [*δ*_H_ 5.20 (1H, d, *J* = 16.4 Hz, H-2), 6.11 (1H, d, *J* = 16.4 Hz, H-3); *δ*_C_ 125.1 (CH, C-2), 141.5 (CH, C-3)] and four oxygenated quaternary carbons [*δ*_C_ 70.9 (qC, C-12), 72.7 (qC, C-4), 75.1 (qC, C-15), 81.1 (qC, C-1)], which accounted for two degrees of unsaturation. The remaining two degrees of unsaturation strongly indicated one bicyclic structure incorporating a nonaromatic oxacycle for **4**. Indeed, compound **4** was identical in all respects with the co-occurring sarcophytrol Q (**9**), which was a diterpene possessing a tetrahydropyran ether across C-1 and C-11 previously isolated from the South China Sea soft coral *S. trocheliophorum* [7]. Their structural difference was in the reversed configuration of C-4, which was deduced from the chemical shift of C-4 upfield, shifting from *δ*_C_ 74.3 in compound **9** to *δ*_C_ 72.7 in compound **4**. This hypothesis was supported by the NOE correlations between H-2 (*δ*_H_ 5.20) and both H_3_-16 (*δ*_H_ 1.16) and H_3_-18 (*δ*_H_ 1.37) implying that H_3_-16 and H_3_-18 were co-facial (Figure 4). After many attempts, suitable single crystals of compound **4** were successfully obtained from the diethyl ether-acetone solvent system to elucidate its absolute configuration. The X-ray diffraction experiment with Ga Kα radiation (*λ* = 1.34139 Å) unambiguously established that the absolute configuration of compound **4** was 1*S*,4*R*,11*R*,12*S* (Flack parameter: −0.06 (9), Figure 5). Based on their epimeric relationship, the absolute configuration of compound **9** could be tentatively assumed to be 1*S*,4*S*,11*R*,12*S*, suggesting the revision of configurations of the C-11 and C-12 of compound **9** reported in the previous work [7].

Sarcoxacyclol E (**5**) had a molecular formula of C_20_H_34_O_5_ deduced from a pseudo-molecular ion peak at *m*/*z* 377.2294 ([M+Na]^+^, calcd. for C_20_H_34_O_5_Na, 377.2298) in the HRESIMS spectrum, requiring four degrees of unsaturation. The ^1^H and ^13^C NMR data of **5** (Table 2) displayed the signals attributed to one disubstituted double bond [*δ*_H_ 5.44 (1H, d, *J* = 15.9 Hz, H-2), 5.75 (1H, d, *J* = 15.9 Hz, H-3); *δ*_C_ 124.1 (CH, C-2), 140.7 (CH, C-3)] and one epoxide group [*δ*_H_ 2.89 (1H, d, *J* = 9.7 Hz, H-7); *δ*_C_ 66.8 (CH, C-7), 61.9 (qC, C-8)]. These two functionalities accounted for two degrees of unsaturation; the left two indicated that compound **5** was a bicyclic diterpene. The interpretation of its ^1^H–^1^H COSY spectrum (Figure 3) disclosed four segments: **a**. H-2–H-3, **b**. H_2_-5–H_2_-6–H-7, **c**. H_2_-9–H_2_-10–H-11, and **d**. H_2_-13–H_2_-14. The HMBC correlations (Figure 3) from H_3_-18 (*δ*_H_ 1.28) to C-3 (*δ*_C_ 140.7), C-4 (*δ*_C_ 74.0), and C-5 (*δ*_C_ 42.4) led to the connection of partial structures **a** and **b** via quaternary carbon C-4 bearing a hydroxyl group. The observed HMBC cross-peaks of H_3_-19 (*δ*_H_ 1.38)/C-7 (*δ*_C_ 66.8), H_3_-19/C-8 (*δ*_C_ 61.9) and H_3_-19/C-9 (*δ*_C_ 35.5) revealed the linkage of partial fragments **b** and **c** through C-8, along with the fixation of the epoxide group at C-7/C-8. The subunit **d** was found to be linked to subunits **c** and **a** by the oxygenated carbons C-12 and C-1, as deduced from the significant HMBC correlations of H_3_-20 (*δ*_H_ 1.17) with C-11 (*δ*_C_ 75.0), C-12 (*δ*_C_ 71.2), and C-13 (*δ*_C_ 37.3), and of both H-2 (*δ*_H_ 5.44) and H-14 (*δ*_H_ 2.22) with C-1 (*δ*_C_ 81.8), respectively. A peculiar isopropyl alcohol group was recognized by the mutual HMBC correlations from H_3_-16 (*δ*_H_ 1.16) and H_3_-17 (*δ*_H_ 1.11) to C-15 (*δ*_C_ 74.6). This group was attached to C-1, as indicated by the key HMBC cross-peaks of H_3_-16/C-1 and H_3_-17/C-1. Thus, the carbon framework of compound **5** was established as a cembrane skeleton. Indeed, the ^13^C chemical shifts of the C-11–C-12–C-13–C-14–C-1 segment of compound **5** were nearly identical to those of the co-isolate **4**, suggesting the hydroxyl group bonded to C-12 and a tetrahydropyran ring bridged C-1 and C-11. Thus, the planar structure of compound **5** was established as an epoxidation derivative of compound **4**. The above-mentioned almost identical chemical shifts of the C-11–C-12–C-13–C-14–C-1 segment suggested the same configurations for the three chiral centers C-1, C-11, and C-12 in compounds **4** and **5**. The NOE correlations (Figure 4) between H-2 and H_3_-17 and between H-3 and H_3_-18, together with the *trans* configuration of double bond Δ^2^ (*J* = 15.9 Hz), suggested H_3_-17 and H_3_-18 were *trans*-oriented. This observation indicated the opposite configuration of C-4 in **5** compared with that of compound **4**, which was further confirmed by the chemical shift of C-4 downshifted from *δ*_C_ 72.7 in compound **4** to *δ*_C_ 74.0 in compound **5**. The diagnostic NOE cross-peak of H-7 (*δ*_H_ 2.89)/H-11 (*δ*_H_ 3.51) implied the co-facial orientation of H-7 and H-11. The *trans* geometry of the epoxide ring was deduced from the NOE cross-peaks of H-7/H-6a (*δ*_H_ 2.10) and H_3_-19/H-6b (*δ*_H_ 1.46). Hereto, the structure of compound **5** was depicted as shown in Figure 1, and the absolute configuration was tentatively assumed to be 1*S*,4*S*,7*S*,8*S*,11*R*,12*S*.

Sarcoxacyclol F (**6**) possessed the same molecular formula (C_20_H_34_O_5_) as that of sarcoxacyclol E (**5**), as deduced from the pseudo-molecular ion peak at *m*/*z* 377.2295 ([M+Na]^+^, calcd. for C_20_H_34_O_5_Na, 377.2298) in the HRESIMS spectrum. Inspection of the NMR data of compound **6** (Table 2) revealed that the planar structure of compound **6** should be the same as that of compound **5**, which was further supported by the extensive analysis of its 2D NMR spectra (Figure 3). The clear NOE cross-peak of H_3_-19 (*δ*_H_ 1.27)/H-11 (*δ*_H_ 3.49) in the NOESY spectrum of compound **6** (Figure 4) suggested the same orientation for CH_3_-19 and H-11 in compound **6**, which was different from that of compound **5**. The *trans* geometry of the epoxide ring in compound **6** was indicated by the key NOE cross-peaks of H-7 (*δ*_H_ 2.95)/H-9a (*δ*_H_ 1.41) and H_3_-19/H-9b (*δ*_H_ 2.03). Consequently, compound **6** was established as the C-7/C-8 stereoisomer of compound **5**, namely another epoxidation derivative of compound **4**, and the absolute configuration was tentatively supposed as 1*S*,4*S*,7*R*,8*R*,11*R*,12*S*.

The origin of compounds **2**, **5,** and **6** is a matter needing discussion. To determine if these three diterpenes were natural products or derived from their co-occurring precursors during the isolation process, we re-checked the crude extract of the soft coral *S. mililatensis* by comparisons of the *R*_f_ values of secondary metabolites in the crude extract with that of pure samples **2**, **5,** and **6** on co-plate TLC as well as comparisons of their retention times on HPLC. We detected the compounds **2**, **5,** and **6** present in the Et_2_O-soluble portion of the acetone extract of the specimen. Evidence from these experiments excluded the possibility that the dehydration and epoxidation derivatives **2**, **5,** and **6** were artifacts obtained during the work-up.

Indeed, some reports have disclosed the coexistence of dehydration derivatives and their related alcohol precursors in one sample, and both of them have been found to be natural products (Figure S49). For instance, Bruce F. Bowden et al. found that the cembrane diterpene 2-[(*E*,*E*,*E*)-7’,8’-epoxy-4’,8’,12’-trimethylcyclotetradeca-1’,3’,11’-trieny1]propan-2-ol (**S1**) coexisted with its dehydration derivative named (*E*,*E*,*E*)-7,8-epoxy-1-isopropenyl-4,8,12-trimethylcyclotetradeca-1,3,11-triene (**S2**) as two natural products in the Australian soft coral *Sarcophyton crassocaule* [32]. Their chemical investigation of another Australian soft coral *Nephthea brassica* led to the discovery of a pair of co-isolates in the sample, namely the dehydration derivative cembrene A (**S3**) and its corresponding alcohol precursor nephthenol (**S4**) [33].

Moreover, several epimeric epoxy analogs were reported as co-occurring isolates in the soft corals (Figure S50). For instance, epimeric epoxy diterpenes sarcocrassolides A (**S5**) and B (**S6**) with close structural similarity coexisted in the South China Sea soft coral *S*. *crassocaule,* as shown in Xu et al.’s work [34]. It might be worthy to point out that the relative configuration of sarcocrassolide B (**S6**) was confirmed by the X-ray diffraction experiment. Sheu et al.’s study on the Formosan soft coral *S*. *crassocaule* also displayed the co-occurrence of epimeric epoxy diterpenes named crassocolides K–M (**S7**–**S9**) [35]. These five cembrane diterpenes are representatives of two groups of diterpenes with epimeric epoxides at C-3/C-4. Seifert et al. have reported the discovery of diterpenes with epimeric epoxides at C-7/C-8, such as dihydrocembrene C (**S10**) and sarcophytoxide (**S11**) from the Indonesian soft coral *Sarcophyton ehrenbergi* [36]. These findings of co-occurring natural epimeric epoxy analogs indicate that there is possibly more than one enzyme to catalyze the epoxidation reaction in the metabolic process in the soft corals, resulting in the seemingly non-stereospecific natural products.

In general, the discovery of the dehydration and epoxidation derivatives in this study inspired us to explore the terpene biosynthetic gene clusters of this species in future, which may be in high demand in addition to the chemo- and bio-investigations.

There have been no reports assessing the tumor necrosis factor (TNF)-*α* inhibitory bioactivity of cembrane diterpenes possessing the tetrahydrofuran and tetrahydropyran ethers. Therefore, all the cembrane diterpenes **1**−**10** were subjected to this bioassay using lipopolysaccharide (LPS)-induced TNF-*α* protein release in RAW264.7 macrophages. As a result, they exhibited different levels of inhibition ratios, ranging from 5.5% to 52.4% at a concentration of 20 μmol/L. Among them, only the new secondary metabolite **1** was judged an active compound (inhibition ratio 52.4%). Further evaluation showed an IC_50_ value of 9.5 μmol/L for compound **1**, which was similar to the positive control dexamethasone (IC_50_ = 8.7 μmol/L). Notably, diterpene **1** displayed no obvious cytotoxic activity towards RAW264.7 cells (CC_50_ > 50 μmol/L). Given the structural differences between compound **1** and its analog compound **2**, it appeared that the isopropyl alcohol moiety at C-1 in the bicyclic cembrane structure played an important role in TNF-*α* inhibitory activity.

**Figure 6:**
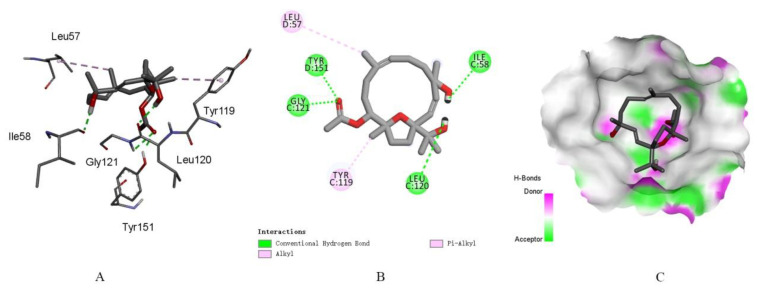
The binding modes of compound **1** at TNF-α crystal structure 2AZ5: (**A**) the clear combination of hydrogen bonds and other interactions within the target pocket; (**B**) two-dimensional ligand interaction diagrams of compound **1** at the crystal structure of the TNF-α protein domain; and (**C**) surfaces of 2AZ5 with the combined compound.

Then, a preliminary molecular docking experiment was performed using the highly resolved TNF-*α* crystal structure (PDB: 2AZ5 with a resolution of 2.10 Å) [37]. As shown in Figure 6, the preliminary computation clearly revealed the hydrogen bonds formed between the hydroxyl group at C-15 and the residue Leu120, between the hydroxyl group at C-4 and the residue Ile58, and between the acetoxyl group at C-11 and the residues Gly121 and Tyr151, which were lying in the active site. Moreover, compound **1** occupied the hydrophobic pocket, where methyl groups at C-7 and C-12 generate π-alkyl stacked interactions with the residues Leu57 and Tyr119. The low binding affinity of compound **1** (−8.02 kcal/mol), which was slightly lower than that of the previously reported ligand 6,7-dimethyl-3-{[methyl-(2-{methyl-[1-(3-trifluoromethyl-phenyl)-1*H*-indol-3-ylmethyl]-amino}-ethyl)-amino]-methyl}-chromen-4-one [37] in the TNF-*α* crystal structure in this study (−5.31 kcal/mol), indicated that compound **1** was a potential TNF-*α* inhibitor.

The study for the elaborated mechanism is in the plan.

## 3. Materials and Methods

### 3.1. General Experimental Procedures

Optical rotations were recorded on a Perkin-Elmer 241MC polarimeter. IR spectra were obtained on a Nicolet 6700 spectrometer (Thermo Scientific, Waltham, MA, USA). CD spectra were measured on a JASCO J-810 instrument. NMR spectra were measured on a Bruker DRX-500 or Bruker DRX-600 spectrometer (Bruker Biospin AG, Fällanden, Germany). Chemical shifts (*δ*) were reported in ppm with reference to the solvent signals, and coupling constants (*J*) were in Hz. ESIMS spectra were obtained on a Finngan-MAT-95 mass spectrometer. HRESIMS spectra were measured on an Agilent 1290-6545 UHPLC-QTOF mass spectrometer. Commercial silica gel (Qingdao Haiyang Chemical Group Co., Ltd., Qingdao, China, 200–300 and 400–600 mesh), Sephadex LH-20 gel (Amersham Biosciences, Piscataway, NJ, USA) were used for column chromatography, and precoated silica gel plates (Yan Tai Zi Fu Chemical Group Co., Yantai, China, G60 F-254) were used for analytical TLC. Reversed-phase (RP) HPLC was performed on an Agilent 1260 series liquid chromatography system equipped with a DAD G1315D detector at 210 and 254 nm. A semi-preparative ODS-HG-5 column [5 µm, 250 × 9.4 mm] was employed for the purifications. All solvents used for column chromatography and HPLC were of analytical grade (Shanghai Chemical Reagents Co., Ltd., Shanghai, China) and chromatographic grade (Dikma Technologies Inc., Foothill Ranch, CA, USA), respectively.

### 3.2. Biological Material

The soft corals of *Sarcophyton mililatensis* were collected at a depth of –20 m by SCUBA diving from the coast of Xigu Island, Hainan Province, China, in May 2014. They were frozen immediately at –20 °C after collection and identified by Prof. X.-B. Li from Hainan University. A voucher specimen (No. 14S-80) is available for inspection at the Shanghai Institute of Materia Medica, Chinese Academy of Sciences.

### 3.3. Extraction and Isolation

The frozen animals (400 g dry weight) were cut into pieces and thoroughly extracted with acetone at room temperature (3 × 1.5 L). The organic extract was evaporated to give a dark brown residue that was partitioned between Et_2_O and H_2_O. The upper layer was concentrated under reduced pressure to give an Et_2_O portion (13.5 g). The Et_2_O extract was separated into twenty-one fractions (A–U) by gradient silica gel column chromatography [0 → 100% Et_2_O (EE) in petroleum ether (PE)]. Fraction G was further purified by Sephadex LH-20 [PE/CH_2_Cl_2_/MeOH (2:1:1)], followed by silica gel column chromatography [PE/EE (4:1)] to give seven subfractions G1–G7. The sixth subfraction G6 was further purified by RP-HPLC [MeCN/H_2_O (85:15), 3.0 mL/min] to give compound **3** (1.8 mg, *t*_R_ = 36.7 min). Similarly, fraction J was further purified by Sephadex LH-20 [PE/CH_2_Cl_2_/MeOH (2:1:1)], followed by silica gel column chromatography [PE/EE (2:1)] to yield seven subfractions J1–J7. Compound **2** (3.6 mg) was isolated from the seventh subfraction J7 by silica gel column chromatography [PE/EE (2:1)]. Through two-step purification, including Sephadex LH-20 [PE/CH_2_Cl_2_/MeOH (2:1:1)] and silica gel column chromatography [PE/ acetone (3:1)], fraction Q was divided into four subfractions, Q1–Q4. The first subfraction Q1 gave compounds **8** (3.1 mg) and **10** (4.3 mg) by silica gel column chromatography [PE/ acetone (7:3)] along with five mixtures Q1A–Q1E. Four mixtures Q1A, Q1B, Q1D, and Q1E yielded compounds **5** (2.5 mg, *t*_R_ = 17.4 min), **6** (2.0 mg, *t*_R_ = 16.9 min), **7** (12.0 mg, *t*_R_ = 10.0 min), and **9** (3.4 mg, *t*_R_ = 9.7 min) by RP-HPLC [MeCN/H_2_O (20:80), 3.0 mL/min; MeCN/H_2_O (40:60), 3.0 mL/min; MeCN/H_2_O (20:80), 3.0 mL/min; MeCN/H_2_O (38:62), 3.0 mL/min], respectively. The second subfraction Q2 yielded compound **1** (8.0 mg, *t*_R_ = 12.5 min) by silica gel column chromatography [PE/acetone (7:3)] followed by RP-HPLC [MeCN/H_2_O (65:35), 3.0 mL/min]. Compound **4** (3.6 mg, *t*_R_ = 4.9 min) was obtained from the fraction R by the two-step purification, including Sephadex LH-20 [PE/CH_2_Cl_2_/MeOH (2:1:1)] and the subsequent RP-HPLC [MeCN/H_2_O (50:50), 3.0 mL/min].

### 3.4. Spectroscopic Data of Compounds

Sarcoxacyclol A (**1**): colorless oil; ${\left[\alpha \right]}_{D}^{25}$ −49.6 (c 0.35, CHCl_3_); IR (KBr): *ν*_max_ 3430, 2971, 2925, 2853, 1732, 1456, 1373, 1241, 1169, 1080, 1028, 983 cm^−1^; For ^1^H NMR (CDCl_3_, 500 MHz) and ^13^C NMR (CDCl_3_, 125 MHz) spectral data, see Table 1; HRESIMS *m/z* 403.2448 ([M+Na]^+^; calcd. for C_22_H_36_NaO_5_, 403.2455).

Sarcoxacyclol B (**2**): colorless oil; ${\left[\alpha \right]}_{D}^{19}$ −36.8 (*c* 0.20, CHCl_3_); IR (KBr): *ν*_max_ 3445, 2969, 2923, 2851, 1733, 1449, 1372, 1238, 1113, 1076, 1027, 964, 899 cm^−1^; For ^1^H NMR (CDCl_3_, 500 MHz) and ^13^C NMR (CDCl_3_, 125 MHz) spectral data, see Table 1; HRESIMS *m/z* 385.2361 ([M+Na]^+^; calcd. for C_22_H_34_NaO_4_, 385.2349).

Sarcoxacyclol C (**3**): colorless oil; ${\left[\alpha \right]}_{D}^{25}$ +4.0 (*c* 0.35, CHCl_3_); IR (KBr): *ν*_max_ 2955, 2923, 2853, 1739, 1457, 1375, 1236, 1132, 1076 cm^−1^; For ^1^H NMR (CDCl_3_, 600 MHz) and ^13^C NMR (CDCl_3_, 125 MHz) spectral data, see Table 1; HREIMS *m/z* 346.2507 (M^+^; calcd. for C_22_H_34_O_3_, 346.2502).

Sarcoxacyclol D (**4**): colorless crystals; mp 183.1-184.0°C; ${\left[\alpha \right]}_{D}^{25}$ +70.1 (*c* 0.35, CHCl_3_); IR (KBr): *ν*_max_ 3440, 2920, 2850, 1463, 1383, 1078, 1047, 947 cm^−1^; For ^1^H NMR (CDCl_3_, 500 MHz) and ^13^C NMR (CDCl_3_, 125 MHz) spectral data, see Table 2; HRESIMS *m/z* 361.2348 (M^+^; calcd. for C_20_H_34_NaO_4_, 361.2349).

Sarcoxacyclol E (**5**): colorless oil; ${\left[\alpha \right]}_{D}^{25}$ +45.8 (*c* 0.35, CHCl_3_); IR (KBr): *ν*_max_ 3440, 2923, 2853, 1462, 1377, 1132 cm^−1^; For ^1^H NMR (CDCl_3_, 600 MHz) and ^13^C NMR (CDCl_3_, 125 MHz) spectral data, see Table 2; HRESIMS *m/z* 377.2294 ([M+Na]^+^; calcd. for C_20_H_34_NaO_5_, 377.2298).

Sarcoxacyclol F (**6**): colorless oil; ${\left[\alpha \right]}_{D}^{25}$ +4.8 (*c* 0.20, CHCl_3_); IR (KBr): *ν*_max_ 3443, 2917, 2849, 1574, 1538, 1457, 1384, 1131, 1049 cm^−1^; For ^1^H NMR (CDCl_3_, 600 MHz) and ^13^C NMR (CDCl_3_, 125 MHz) spectral data, see Table 2; HRESIMS *m/z* 377.2295 ([M+Na]^+^; calcd. for C_20_H_34_NaO_5_, 377.2298).

### 3.5. Crystal Structure Analysis of ***4***

Block crystals of compound **4** were obtained from diethyl ether-acetone solvent systems at 4 °C. Single-crystal X-ray diffraction of compound **4** (0.16 × 0.11 × 0.10 mm^3^) was performed on a Bruker D8 Venture diffractometer using Ga Kα radiation (*λ* = 1.34139 Å) at 170 K. The collected data integration and reduction were processed with SAINT software (V8.37A, **2015**, Bruker AXS Inc., Karlsruhe, Germany), and multi-scan absorption corrections were performed using the SADABS program (V2016/2, **2016**, Bruker AXS Inc., Karlsruhe, Germany). The structures were solved by direct methods using SHELXL (V2016/6, **2016**, George M. Sheldrick) and refined on *F*^2^ by the full-matrix least-squares technique using the SHELXT-2015 program package. Crystallographic data for compound **4** in this article (Table S1) have been deposited at the Cambridge Crystallographic Data Centre (deposition numbers CCDC 2225113). Copies of these data can be obtained free of charge via www.ccdc.cam.ac.uk/conts/retrieving.html (accessed on 8 December 2022) or from the Cambridge Crystallographic Data Centre (12 Union Road, Cambridge CB21EZ, UK. Fax: (+44) 1223-336-033. E-mail: deposit@ccdc.cam.ac.uk).

### 3.6. TNF-α Inhibitory Activity Bioassay

A murine macrophage cell line, RAW264.7 cells, was obtained from the American Type Culture Collection (ATCC, Manassas, VA, USA). In the bioassay for anti-inflammation, cells were cultured in DMEM containing 10% FBS, 2 mmol/L L-glutamine, 100 μg/mL streptomycin, and 100 U/mL penicillin in a humidified incubator of 5% CO_2_ at 37 °C. For the cytotoxicity part, RAW264.7 cells were incubated with the compounds or the media (0.125% DMSO in DMEM containing 10% FBS) for 24h, respectively. CCK-8 reagents (20 μL per well) were added, and the OD values were collected after 1h incubation at 450 nm (650 nm calibration) by a microplate reader (Molecular Devices, Sunnyvale, CA, USA). For the anti-inflammatory activity assay, RAW264.7 cells were incubated with compounds or the media (0.125% DMSO in DMEM containing 10% FBS), and then cells were primed with LPS (1 μg/mL) for 24 h. The supernatants were centrifuged and then measured using the mouse TNF-α ELISA kit. The CC_50_ and IC_50_ were estimated using the log (inhibitor) vs. normalized response non-linear fit (Graph Pad Prism 6.0, GraphPad Software, San Diego, CA, USA). Dexamethasone was used as a positive control.

### 3.7. ECD Computational Protocol

Torsional sampling (MCMM) conformational searches using the OPLS_2005 force field were carried out by means of the conformational search module in the Macromodel 9.9.223 software [38]*,* applying an energy window of 21 kJ/mol. Conformers above 1% Boltzmann populations were reoptimized with Gaussian 09 [39] at the B3LYP/6-311G(d,p) level with the IEFPCM solvent model for chloroform. Frequency analysis was also carried out to confirm that the reoptimized geometries were at the energy minima. Finally, the SpecDis1.62 software [40] was applied to obtain the Boltzmann-averaged ECD spectra and visualize the results.

### 3.8. Molecular Docking

The docking study was performed using the Operation Environment (ADT 1.5.7) software (https://autodock.scripps.edu/, accessed on 20 August 2022) between the compound and TNF-α (PDB: 2AZ5). The structure of the natural product was optimized by energy minimization using the MM2 method and converted to a readable format at the ADT interface. Replication of the experimental binding posed by molecular docking confirmed the suitability of the docking algorithm (RMSD <2 Å). The outcomes of the results were analyzed using the Discovery Studio Visualizer software, which reveals close contact, hydrogen bond, and hydrophobic interactions. The binding affinity of the known ligand 6,7-dimethyl-3-{[methyl-(2-{methyl-[1-(3-trifluoromethyl-phenyl)-1*H*-indol-3-ylmethyl]-amino}-ethyl)-amino]-methyl}-chromen-4-one [37] in the TNF-α crystal structure was −5.31 kcal/mol.

## 4. Conclusions

The present study provided the intriguing discovery of ten cembrane diterpenes from the Hainan soft coral *S. mililatensis*, which was rarely documented in literature. Among these secondary metabolites, six were new compounds. Their structures were elucidated by extensive spectroscopic analysis along with a comparison with existing data in the literature. Different types of nonaromatic oxacycles were found in their structures, which were identified as tetrahydrofuran and tetrahydropyran ethers that rarely occurred between C-1 and C-12 and between C-1 and C-11, respectively. Additionally, the absolute configuration of compound **4** was established unambiguously by an X-Ray diffraction experiment. The absolute configuration of **7** was assigned by the modified Mosher’s method in our previous work [7]. Based on the comparison of ECD spectra, the absolute configuration of compound **8** was revealed in the present study. Subsequently, the absolute configurations of the other five new compounds were tentatively assumed using the diterpenes **4**, **7** and **8** as model compounds. In the bioassay, the new secondary metabolite, compound **1,** exhibited significant TNF-*α* inhibitory activity with an IC_50_ value of 9.5 μmol/L and was not cytotoxic towards RAW264.7 cells (CC_50_ > 50 μmol/L). A molecular docking experiment gave insight into the key binding actions between the active compound **1** and TNF-*α*, which indicated the important roles of the hydroxyl groups at C-4 and C-15 and the acetoxyl group at C-11. Although the elaborated mechanism is still under investigation, the present preliminary pharmacological result revealed that the natural product, compound **1,** could be developed as a potential lead compound or drug candidate of a new chemotype of nontoxic TNF-*α* inhibitors. This study not only extends the chemical and biological diversities of cembrane diterpenes possessing nonaromatic oxacycles but also establishes the productivity of the title species.

## Figures and Tables

**Table 1:** ^1^H NMR and ^13^C NMR data for compounds **1–3**.

No.	1 ^a^		2 ^a^		3 ^b^	
	*δ*_H_ mult. (*J*, Hz)	*δ*_C_, type	*δ*_H_ mult. (*J*, Hz)	*δ*_C_, Type	*δ*_H_ mult. (*J*, Hz)	*δ*_C_, Type
1		91.4, qC		88.1, qC		89.6, qC
2	5.55 d (15.8)	129.8, CH	5.68 d (15.5)	130.4, CH	2.14 m; 2.35 m	35.7, CH_2_
3	6.11 d (15.8)	137.4, CH	5.92 d (15.5)	136.4, CH	5.12 t (6.0)	119.9, CH
4		72.4, qC		74.1, qC		136.6, qC
5	1.69 m; 1.86 m	41.6, CH_2_	1.77 m; 1.82 m	43.5, CH_2_	2.21 m	38.0, CH_2_
6	2.01 m; 2.63 m	22.9, CH_2_	2.20 m	24.3, CH_2_	2.22 m	24.9, CH_2_
7	5.42 dd (4.3, 10.5)	130.1, CH	5.37 dd (6.2, 8.8)	128.3, CH	5.18 t (6.0)	126.2, CH
8		132.1, qC		132.6, qC		132.9, qC
9	1.86 m; 2.00 m	34.8, CH_2_	1.76 m; 2.00 m	34.2, CH_2_	1.67 m; 2.02 m	33.8, CH_2_
10	1.54 m; 1.89 m	27.3, CH_2_	1.56 m; 1.96 m	27.3, CH_2_	1.53 m; 1.85 m	27.5, CH_2_
11	5.07 d (9.4)	77.1, CH	5.01 d (9.7)	76.5, CH	4.88 d (10.2)	75.7, CH
12		84.5, qC		84.2, qC		83.5, qC
13	1.58 m; 1.74 m	35.8, CH_2_	1.59 m; 1.78 m	35.3, CH_2_	1.55 m; 1.77 m	35.3, CH_2_
14	1.68 m; 2.31 m	30.8, CH_2_	2.03 m	35.3, CH_2_	1.78 m; 1.93 m	32.0, CH_2_
15		72.9, qC		149.5, qC		150.2, qC
16	1.12 s	24.6, CH_3_	4.71 s; 4.96 s	109.4, CH_2_	4.80 s; 5.10 s	110.1, CH_2_
17	1.10 s	26.0, CH_3_	1.71 s	19.2, CH_3_	1.75 s	20.1, CH_3_
18	1.34 s	29.0, CH_3_	1.28 s	28.6, CH_3_	1.56 s	16.8, CH_3_
19	1.70 s	16.3, CH_3_	1.66 s	17.1, CH_3_	1.63 s	17.6, CH_3_
20	1.16 s	20.7, CH_3_	1.18 s	21.0, CH_3_	1.18 s	21.8, CH_3_
OAc		171.2, qC		171.2, qC		171.3, qC
	2.00 s	21.4, CH_3_	2.05 s	21.4, CH_3_	2.06 s	21.5, CH_3_

^a^ Recorded at 500 and 125 MHz for ^1^H and ^13^C in CDCl_3_, respectively. ^b^ Recorded at 600 and 125 MHz for ^1^H and ^13^C in CDCl_3_, respectively. Assignments were deduced by analyzing 1D and 2D NMR spectra.

**Table 2:** ^1^H NMR and ^13^C NMR data for compounds **4–6**.

No.	4 ^a^		5 ^b^		6 ^b^	
	*δ*_H_ mult. (*J*, Hz)	*δ*_C_, Type	*δ*_H_ mult. (*J*, Hz)	*δ*_C_, type	*δ*_H_ mult. (*J*, Hz)	*δ*_C_, Type
1		81.1, qC		81.8, qC		80.3, qC
2	5.20 d (16.4)	125.1, CH	5.44 d (15.9)	124.1, CH	5.53 d (16.0)	124.4, CH
3	6.11 d (16.4)	141.5, CH	5.75 d (15.9)	140.7, CH	6.26 d (16.0)	142.8, CH
4		72.7, qC		74.0, qC		73.6, qC
5	1.55 m; 1.93 m	43.3, CH_2_	1.76 m; 2.03 m	42.4, CH_2_	1.86 m	38.0, CH_2_
6	2.06 m; 2.58 m	22.9, CH_2_	2.08 m; 2.30 m	24.2, CH_2_	1.59 m; 2. 02 m	25.0, CH_2_
7	5.23 dd (5.1, 10.2)	131.0, CH	2.89 d (9.7)	66.8, CH	2.95 dd (4.2, 9.0)	67.0, CH
8		132.6, qC		61.9, qC		60.3, qC
9	1.51 m; 1.70 m	39.0, CH_2_	1.41 m; 2.15 m	35.5, CH_2_	1.41 m; 2.03 m	33.3, CH_2_
10	1.42 m; 1.88 m	26.6, CH_2_	1.42 m; 1.87 m	25.0, CH_2_	1.46 m; 1.90 m	24.4, CH_2_
11	3.35 t (4.1)	75.6, CH	3.51 t (5.1)	75.0, CH	3.49 t (10.8)	74.1, CH
12		70.9, qC		71.2, qC		70.3, qC
13	2.01 m; 2.21 m	37.3, CH_2_	1.53 m; 1.70 m	37.3, CH_2_	1.71 m	36.7, CH_2_
14	1.54 m; 2.16 m	28.5, CH_2_	1.60 m; 2.22 m	28.2, CH_2_	1.61 m; 2.12 m	28.2, CH_2_
15		75.1, qC		74.6, qC		75.0, qC
16	1.16 s	24.7, CH_3_	1.16 s	24.8, CH_3_	1.17 s	24.7, CH_3_
17	1.09 s	24.1, CH_3_	1.11 s	24.0, CH_3_	1.14 s	24.4, CH_3_
18	1.37 s	28.0, CH_3_	1.28 s	27.7, CH_3_	1.39 s	27.2, CH_3_
19	1.70 s	15.2, CH_3_	1.38 s	17.8, CH_3_	1.27 s	15.5, CH_3_
20	1.18 s	19.6, CH_3_	1.17 s	19.2, CH_3_	1.16 s	19.7, CH_3_

^a^ Recorded at 500 and 125 MHz for ^1^H and ^13^C in CDCl_3_, respectively. ^b^ Recorded at 600 and 125 MHz for ^1^H and ^13^C in CDCl_3_, respectively. Assignments were deduced by analyzing 1D and 2D NMR spectra.

## Data Availability

The Data are contained within the article or Supplementary Materials.

## Supplementary Materials

The following supporting information can be downloaded at: https://www.mdpi.com/article/10.3390/ijms24031979/s1, Figures S1–S48: MS, NMR, and IR data of compounds **1**–**6**; Figure S49: Examples of the coexistence of dehydration derivatives and their related alcohol precursors from different soft corals; Figure S50: Examples of the coexistence of epimeric epoxy analogs from different soft corals; Table S1: X-ray crystallographic data for compound **4**.
