# Supplementary material for: Cembrane Diterpenes Possessing Nonaromatic Oxacycles from the Hainan Soft Coral Sarcophyton mililatensis
Source: Int J Mol Sci. 2023 Jan 19;24(3):1979. doi: 10.3390/ijms24031979 (PMC9915928; doi:10.3390/ijms24031979)
Supplement: Supplementary file 1 [file ijms-24-01979-s001.zip › ijms-2136998-supplementary.pdf]

Supplementary Information

Cembrane Diterpenes Encompassed Nonaromatic Oxacycles from the Hainan Soft Coral *Sarcophyton mililatensis*

Table of contents

**Figure S1.**	HRESIMS spectrum of compound **1**
**Figure S2.**	^{1}H NMR spectrum (500 MHz) of compound **1** in $CDCl_3$
**Figure S3.**	^{13}C NMR (BB+DEPT) spectrum (125 MHz) of compound **1** in $CDCl_3$
**Figure S4.**	HSQC spectrum (500 MHz) of compound **1** in $CDCl_3$
**Figure S5.**	^{1}H–^{1}H COSY spectrum (500 MHz) of compound **1** in $CDCl_3$
**Figure S6.**	HMBC spectrum (500 MHz) of compound **1** in $CDCl_3$
**Figure S7.**	NOESY spectrum (500 MHz) of compound **1** in $CDCl_3$
**Figure S8.**	IR spectrum of compound **1**
**Figure S9.**	HRESIMS spectrum of compound **2**
**Figure S10.**	^{1}H NMR spectrum (500 MHz) of compound **2** in $CDCl_3$
**Figure S11.**	^{13}C NMR (BB+DEPT) spectrum (125 MHz) of compound **2** in $CDCl_3$
**Figure S12.**	HSQC spectrum (500 MHz) of compound **2** in $CDCl_3$
**Figure S13.**	^{1}H–^{1}H COSY spectrum (500 MHz) of compound **2** in $CDCl_3$
**Figure S14.**	HMBC spectrum (500 MHz) of compound **2** in $CDCl_3$
**Figure S15.**	NOESY spectrum (500 MHz) of compound **2** in $CDCl_3$
**Figure S16.**	IR spectrum of compound **2**
**Figure S17.**	HRESIMS spectrum of compound **3**
**Figure S18.**	^{1}H NMR spectrum (600 MHz) of compound **3** in $CDCl_3$
**Figure S19.**	^{13}C NMR (BB+DEPT) spectrum (125 MHz) of compound **3** in $CDCl_3$
**Figure S20.**	HSQC spectrum (600 MHz) of compound **3** in $CDCl_3$
**Figure S21.**	^{1}H–^{1}H COSY spectrum (600 MHz) of compound **3** in $CDCl_3$
**Figure S22.**	HMBC spectrum (600 MHz) of compound **3** in $CDCl_3$
**Figure S23.**	NOESY spectrum (600 MHz) of compound **3** in $CDCl_3$
**Figure S24.**	IR spectrum of compound **3**
**Figure S25.**	HRESIMS spectrum of compound **4**
**Figure S26.**	^{1}H NMR spectrum (500 MHz) of compound **4** in $CDCl_3$
**Figure S27.**	^{13}C NMR (BB+DEPT) spectrum (125 MHz) of compound **4** in $CDCl_3$
**Figure S28.**	HSQC spectrum (500 MHz) of compound **4** in $CDCl_3$
**Figure S29.**	^{1}H–^{1}H COSY spectrum (500 MHz) of compound **4** in $CDCl_3$
**Figure S30.**	HMBC spectrum (500 MHz) of compound **4** in $CDCl_3$
**Figure S31.**	NOESY spectrum (500 MHz) of compound **4** in $CDCl_3$
**Figure S32.**	IR spectrum of compound **4**
**Figure S33.**	HRESIMS spectrum of compound **5**
**Figure S34.**	^{1}H NMR spectrum (600 MHz) of compound **5** in $CDCl_3$
**Figure S35.**	^{13}C NMR (BB+DEPT) spectrum (125 MHz) of compound **5** in $CDCl_3$
**Figure S36.**	HSQC spectrum (600 MHz) of compound **5** in $CDCl_3$
**Figure S37.**	^{1}H–^{1}H COSY spectrum (600 MHz) of compound **5** in $CDCl_3$
**Figure S38.**	HMBC spectrum (600 MHz) of compound **5** in $CDCl_3$
**Figure S39.**	NOESY spectrum (600 MHz) of compound **5** in $CDCl_3$
**Figure S40.**	IR spectrum of compound **5**
**Figure S41.**	HRESIMS spectrum of compound **6**
**Figure S42.**	^{1}H NMR spectrum (600 MHz) of compound **6** in $CDCl_3$
**Figure S43.**	^{13}C NMR (BB+DEPT) spectrum (125 MHz) of compound **6** in $CDCl_3$
**Figure S44.**	HSQC spectrum (600 MHz) of compound **6** in $CDCl_3$
**Figure S45.**	^{1}H–^{1}H COSY spectrum (600 MHz) of compound **6** in $CDCl_3$
**Figure S46.**	HMBC spectrum (600 MHz) of compound **6** in $CDCl_3$
**Figure S47.**	NOESY spectrum (600 MHz) of compound **6** in $CDCl_3$
**Figure S48.**	IR spectrum of compound **6**
**Figure S49.**	Examples of the coexistence of dehydration derivatives and their related alcohol precursors from different soft corals.
**Figure S50.**	Examples of the coexistence of epimeric epoxy analogues from different soft corals.
**Table S1.**	X-ray crystallographic data for compound **4**

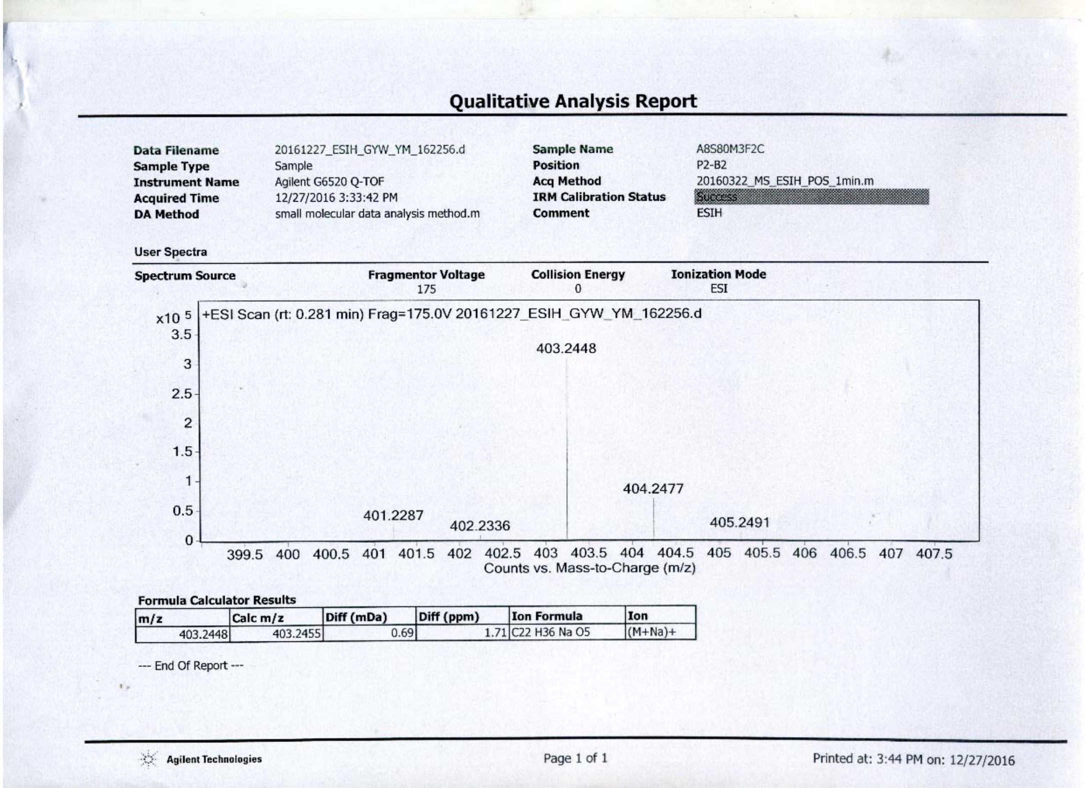

Qualitative Analysis Report

**Data Filename**	20161227_ESIH_GYW_YM_162256.d	**Sample Name**	A8S80M3F2C
**Sample Type**	Sample	**Position**	P2-B2
**Instrument Name**	Agilent G6520 Q-TOF	**Acq Method**	20160322_MS_ESIH_POS_1min.m
**Acquired Time**	12/27/2016 3:33:42 PM	**IRM Calibration Status**	Success
**DA Method**	small molecular data analysis method.m	**Comment**	ESIH

User Spectra

Spectrum Source	Fragmentor Voltage	Collision Energy	Ionization Mode
	175	0	ESI

Formula Calculator Results

m/z	Calc m/z	Diff (mDa)	Diff (ppm)	Ion Formula	Ion
403.2448	403.2455	0.69	1.71	C22 H36 Na O5	(M+Na)+

--- End Of Report ---

Agilent Technologies Page 1 of 1 Printed at: 3:44 PM on: 12/27/2016

Figure S1. HRESIMS spectrum of compound **1**

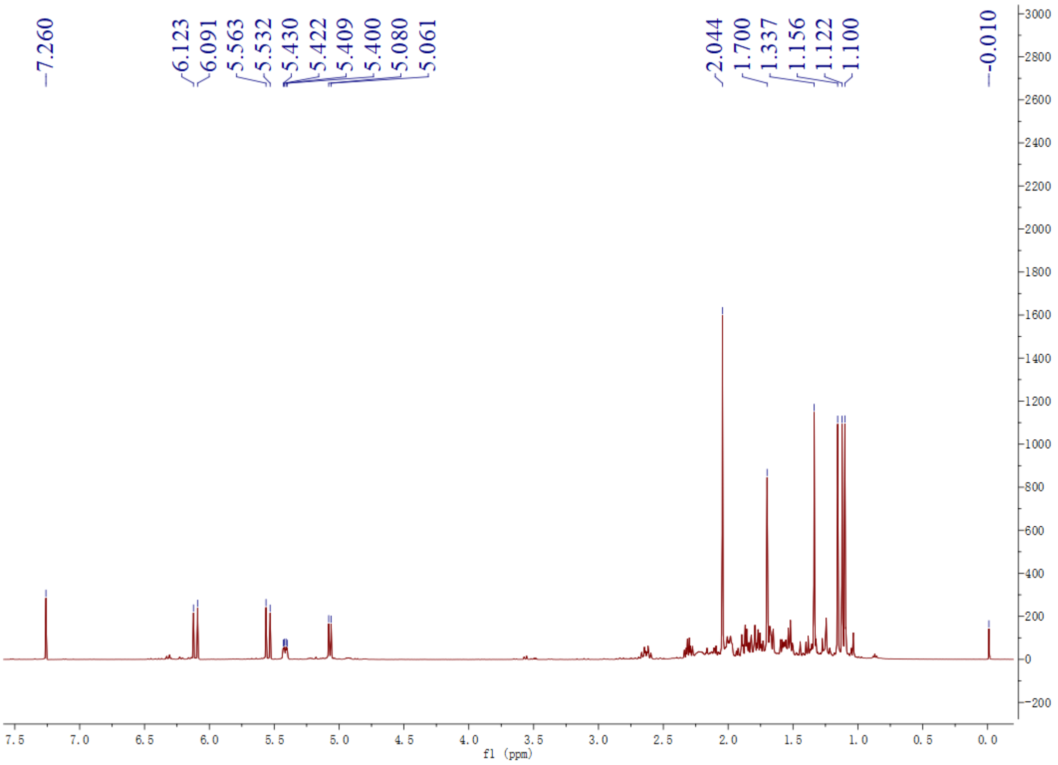

Figure S2. ^{1}H NMR spectrum (500 MHz) of compound **1** in $CDCl_3$

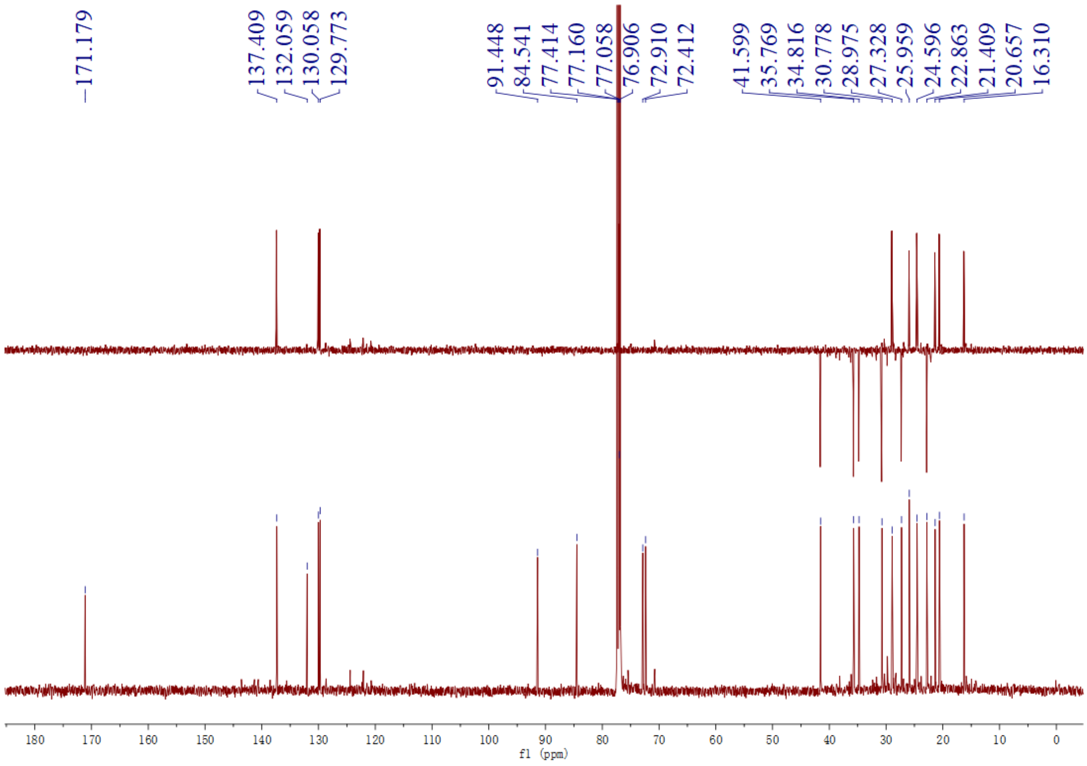

Figure S3. ^{13}C NMR (BB+DEPT) spectrum (125 MHz) of compound **1** in $CDCl_3$

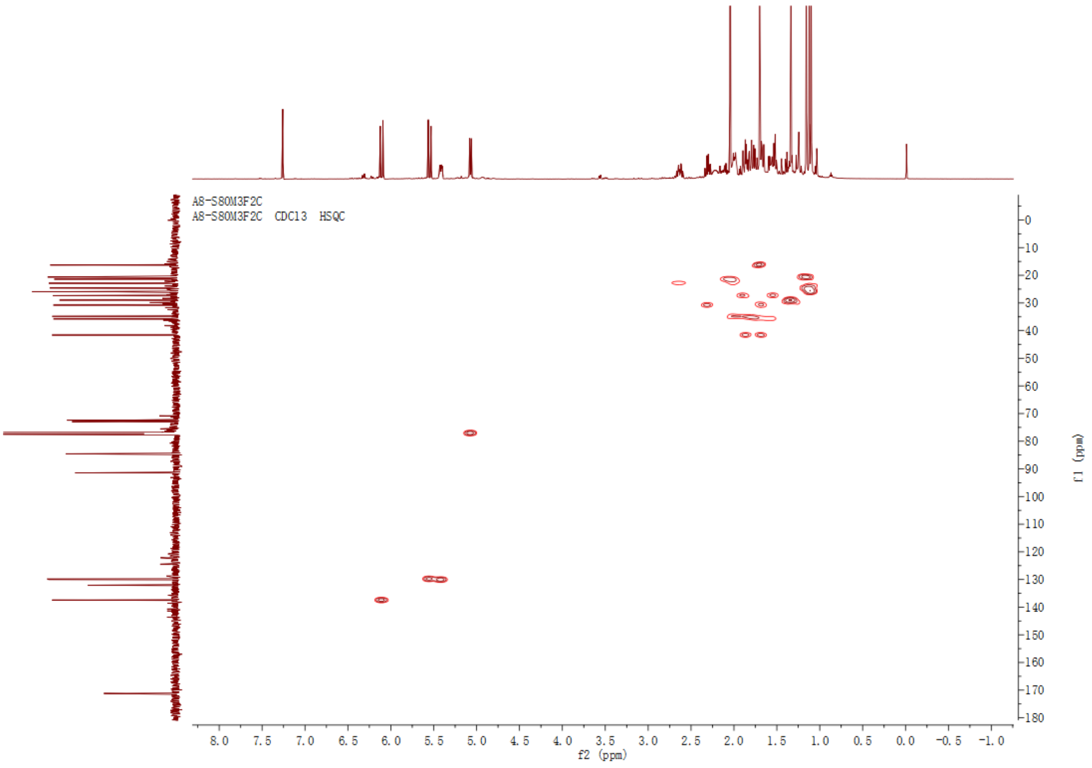

Figure S4. HSQC spectrum (500 MHz) of compound **1** in $CDCl_3$

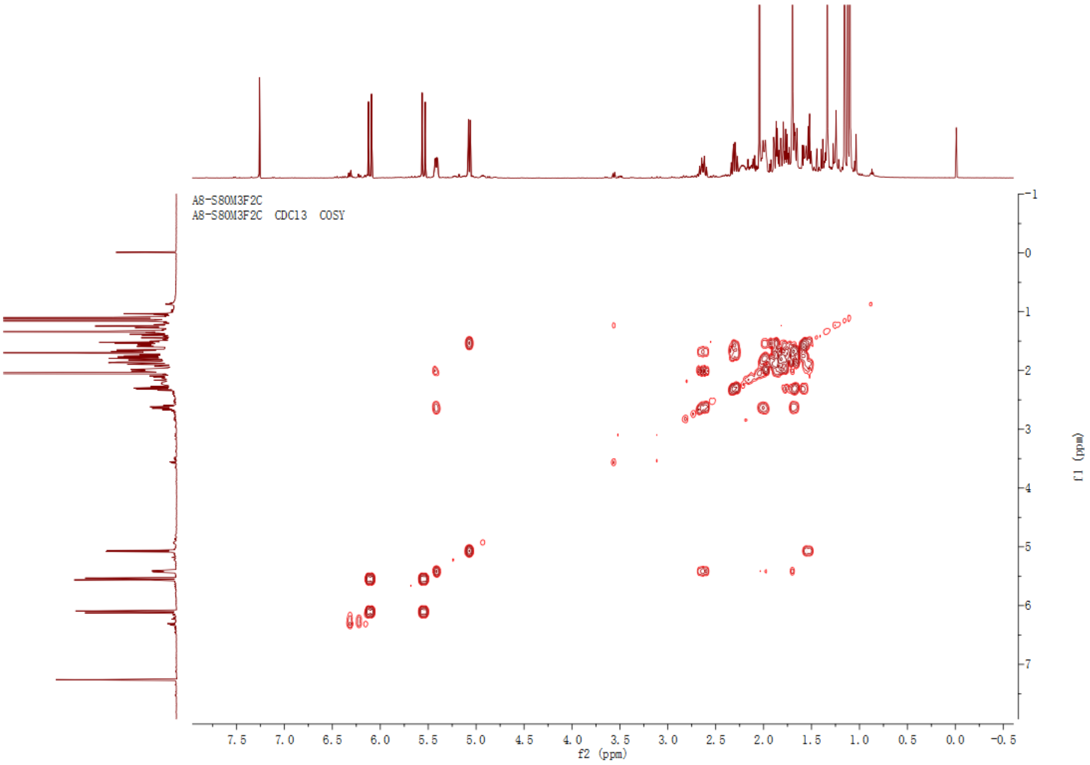

Figure S5. ^{1}H–^{1}H COSY spectrum (500 MHz) of compound **1** in $CDCl_3$

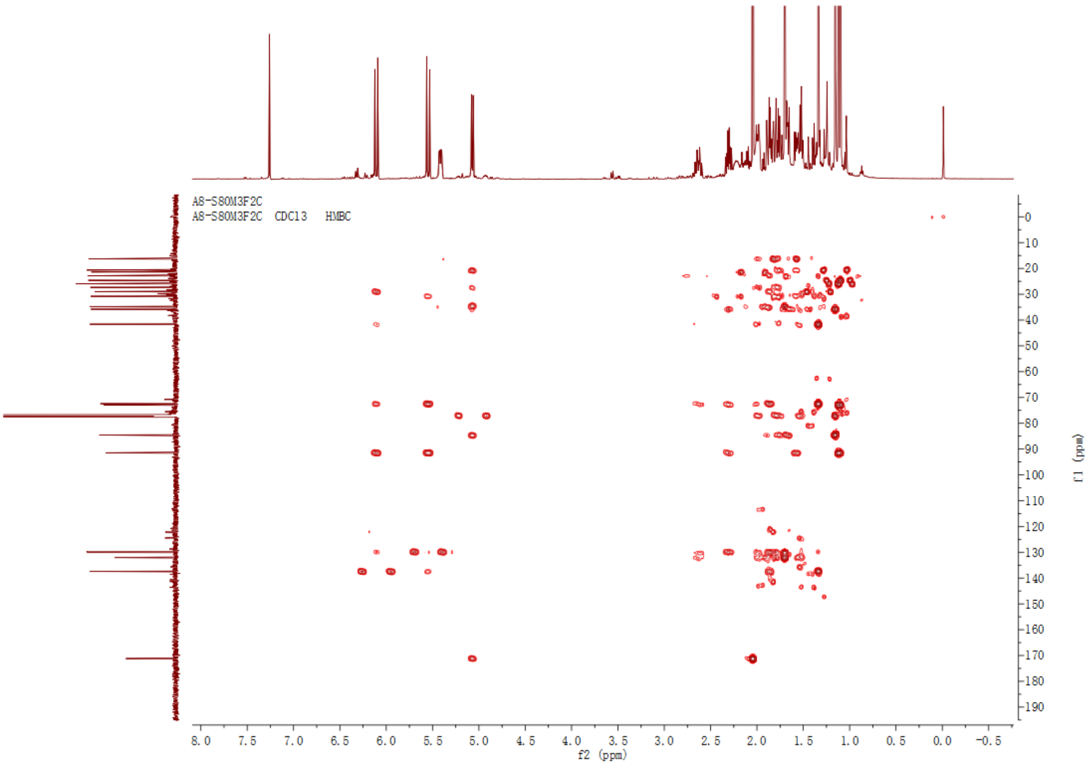

Figure S6. HMBC spectrum (500 MHz) of compound **1** in $CDCl_3$

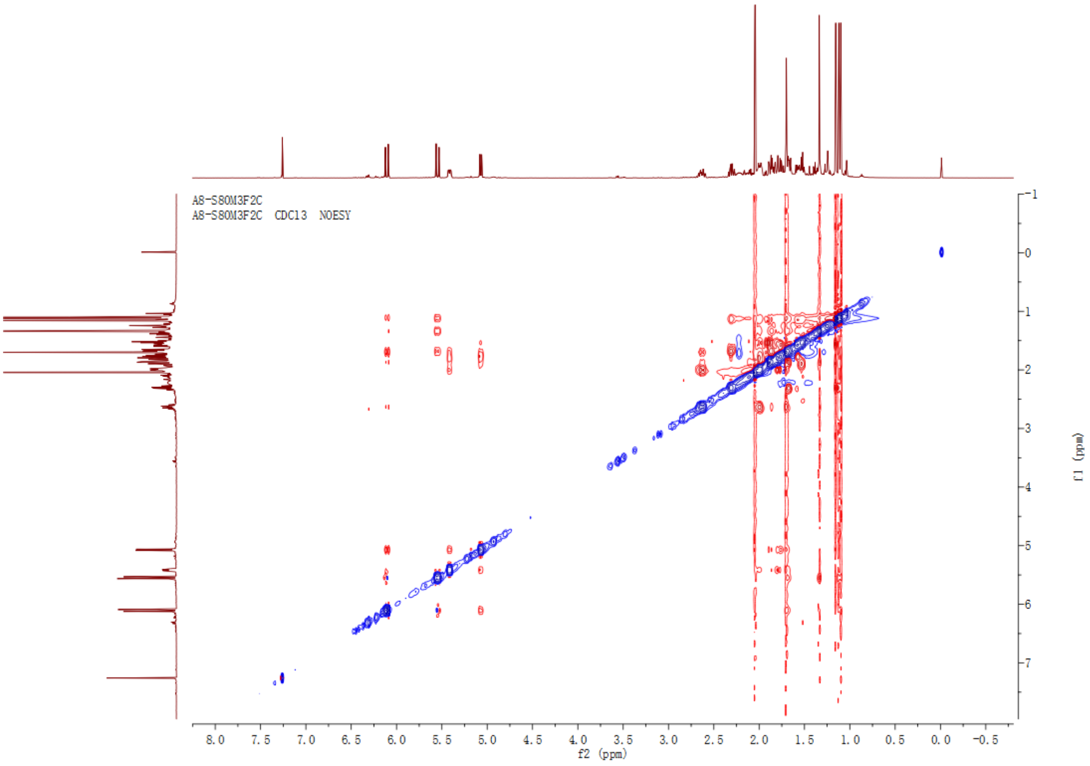

Figure S7. NOESY spectrum (500 MHz) of compound **1** in $CDCl_3$

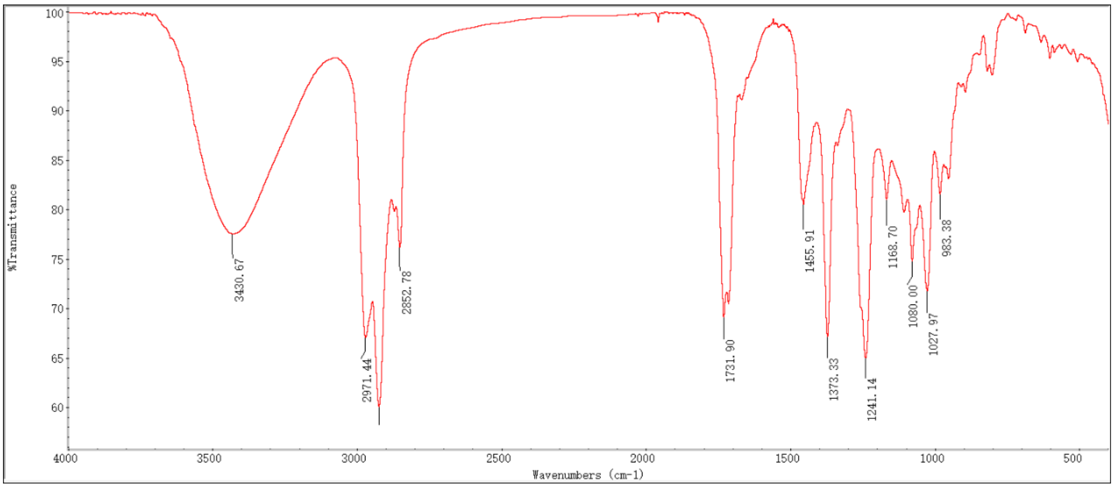

Figure S8. IR spectrum of compound **1**

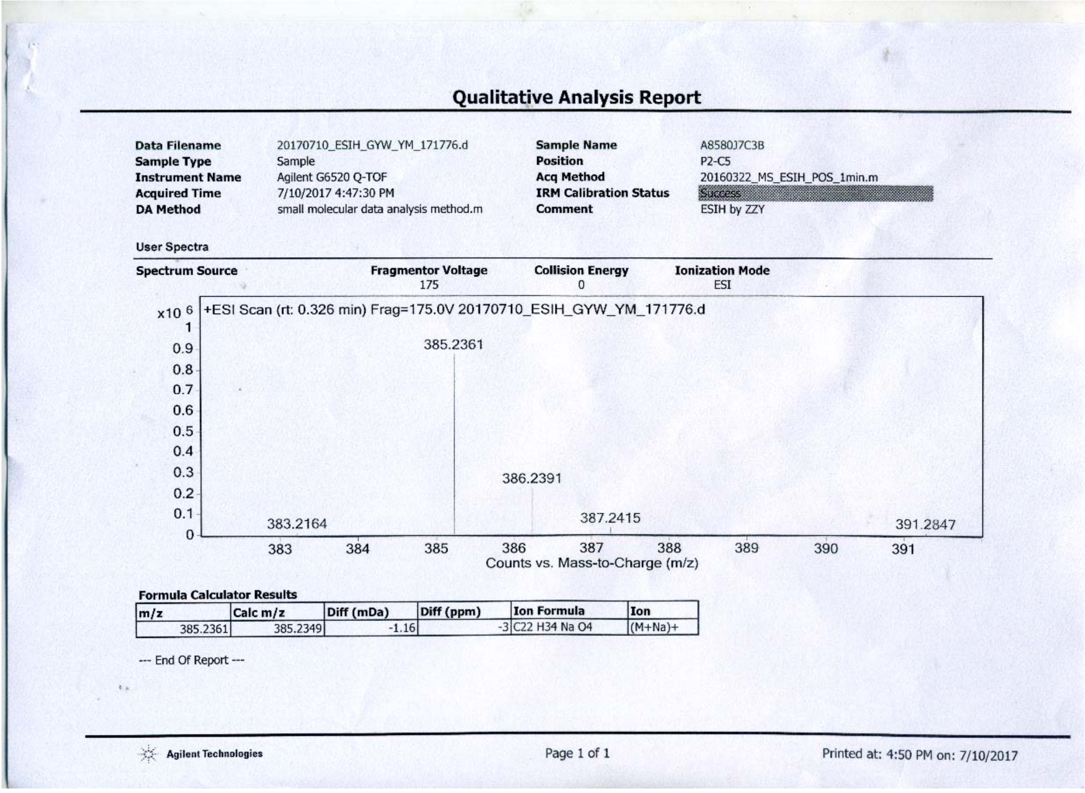

Qualitative Analysis Report

**Data Filename**	20170710_ESIH_GYW_YM_171776.d	**Sample Name**	A858OJ7C3B
**Sample Type**	Sample	**Position**	P2-C5
**Instrument Name**	Agilent G6520 Q-TOF	**Acq Method**	20160322_MS_ESIH_POS_1min.m
**Acquired Time**	7/10/2017 4:47:30 PM	**IRM Calibration Status**	Success
**DA Method**	small molecular data analysis method.m	**Comment**	ESIH by ZZY

User Spectra

Spectrum Source	Fragmentor Voltage	Collision Energy	Ionization Mode
	175	0	ESI

Formula Calculator Results

m/z	Calc m/z	Diff (mDa)	Diff (ppm)	Ion Formula	Ion
385.2361	385.2349	-1.16	-3	C22 H34 Na O4	(M+Na)+

--- End Of Report ---

Agilent Technologies Page 1 of 1 Printed at: 4:50 PM on: 7/10/2017

Figure S9. HRESIMS spectrum of compound **2**

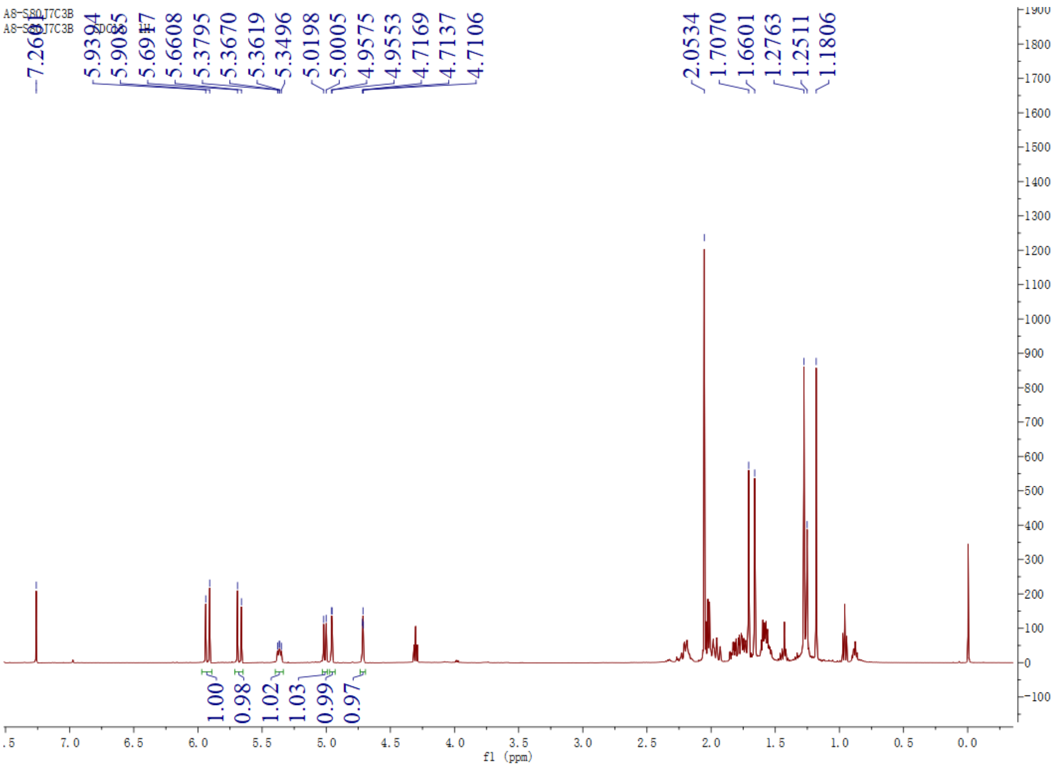

Figure S10. ^{1}H NMR spectrum (500 MHz) of compound **2** in $CDCl_3$

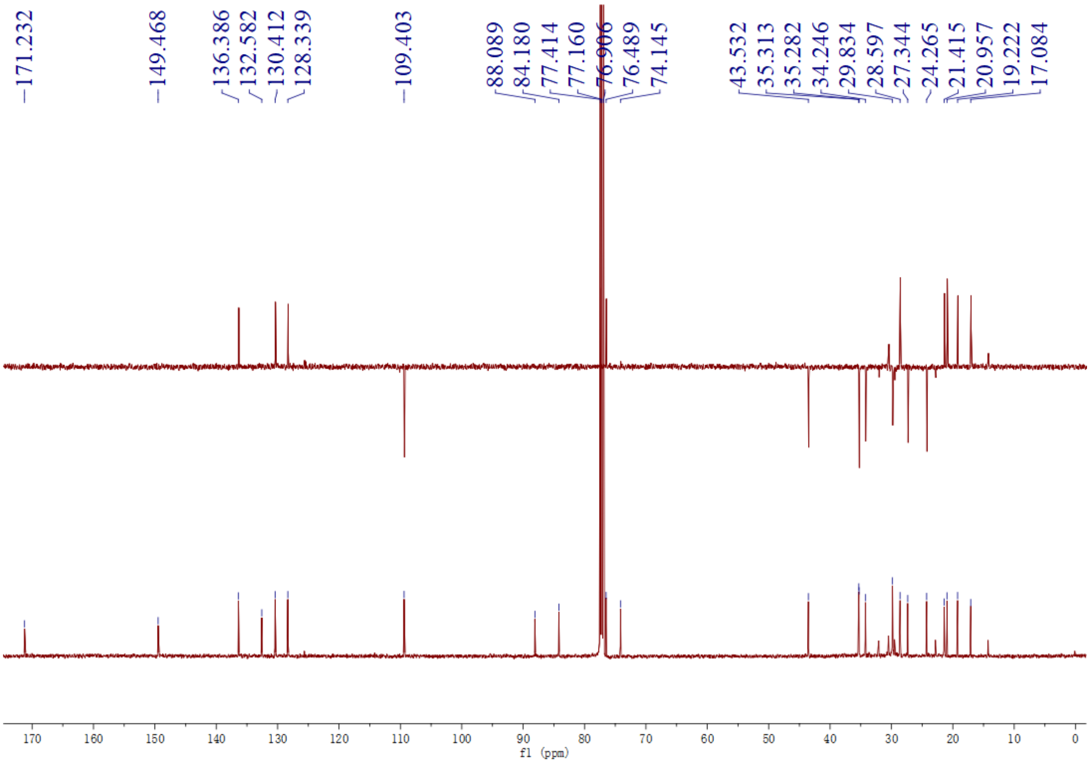

Figure S11. ^{13}C NMR (BB+DEPT) spectrum (125 MHz) of compound **2** in $CDCl_3$

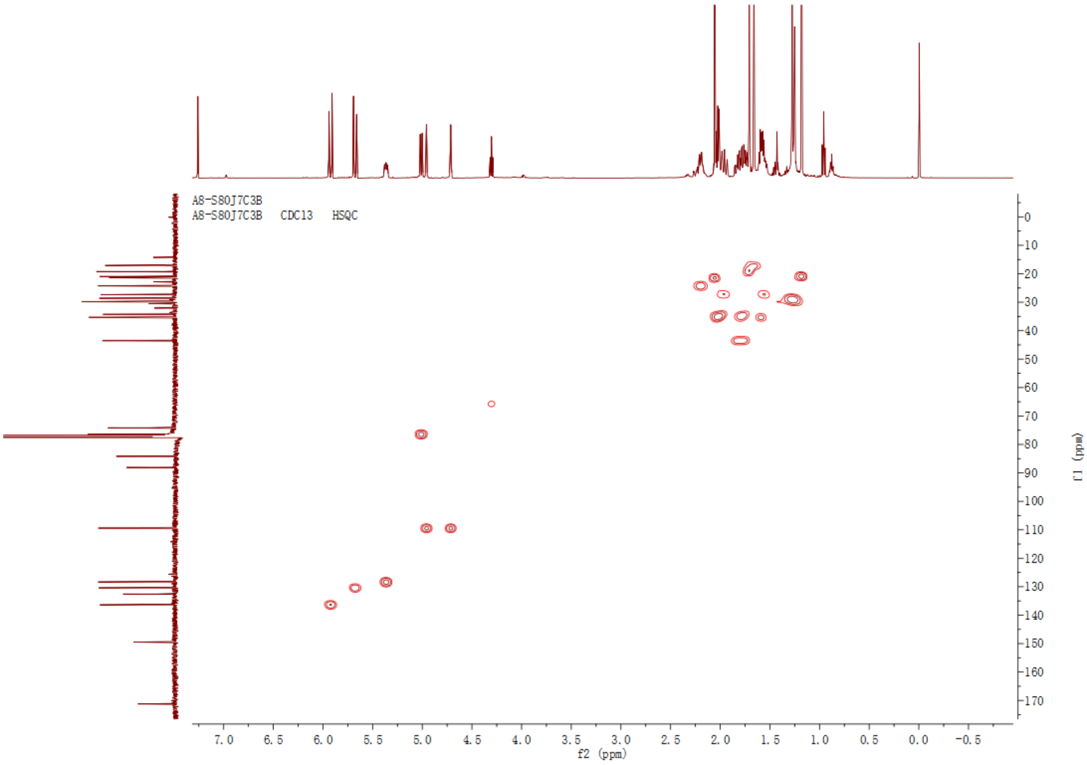

Figure S12. HSQC spectrum (500 MHz) of compound **2** in $CDCl_3$

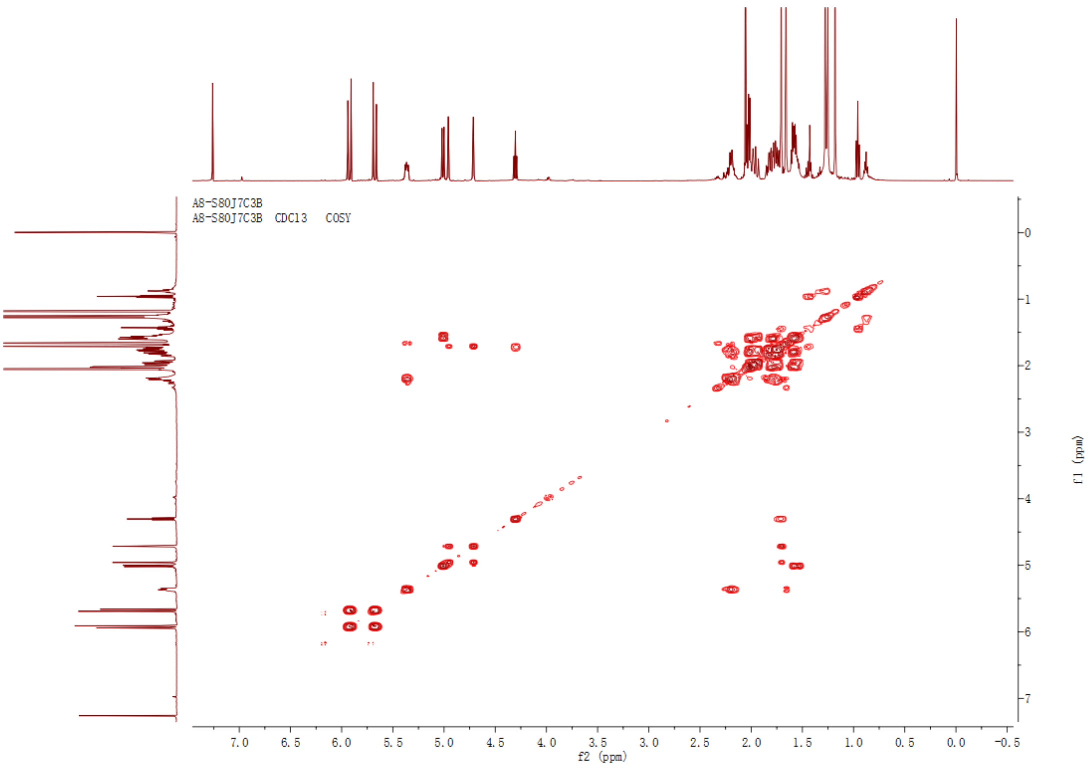

Figure S13. ^{1}H–^{1}H COSY spectrum (500 MHz) of compound **2** in $CDCl_3$

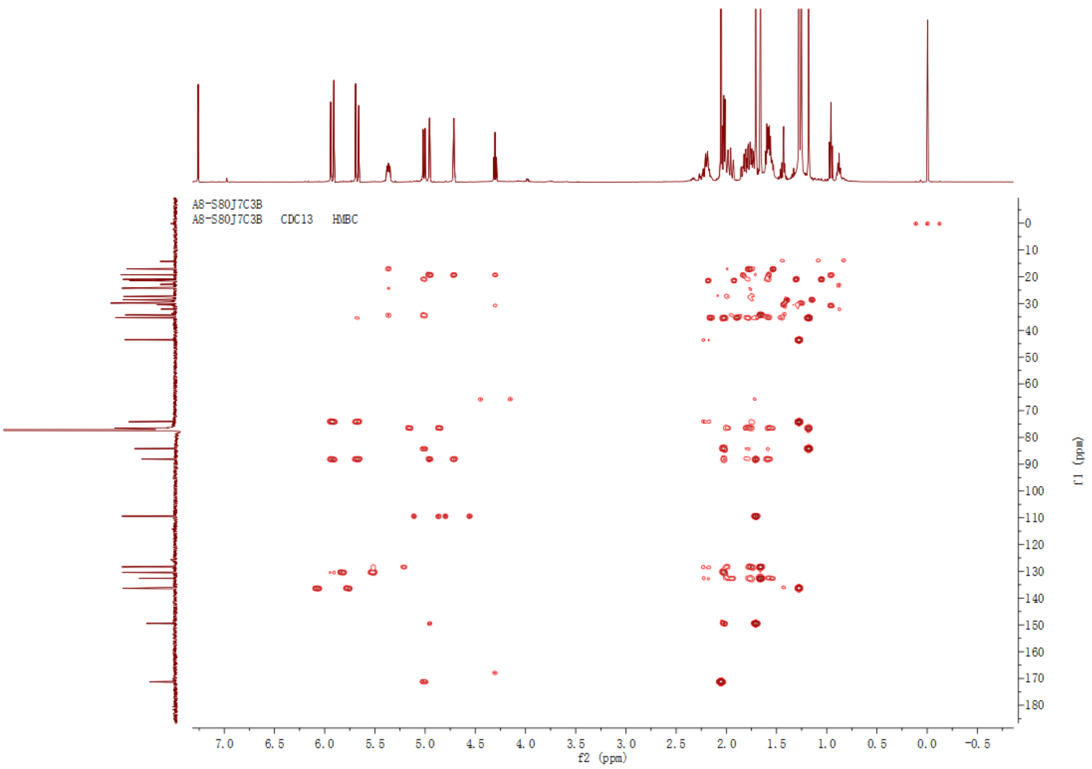

Figure S14. HMBC spectrum (500 MHz) of compound **2** in $CDCl_3$

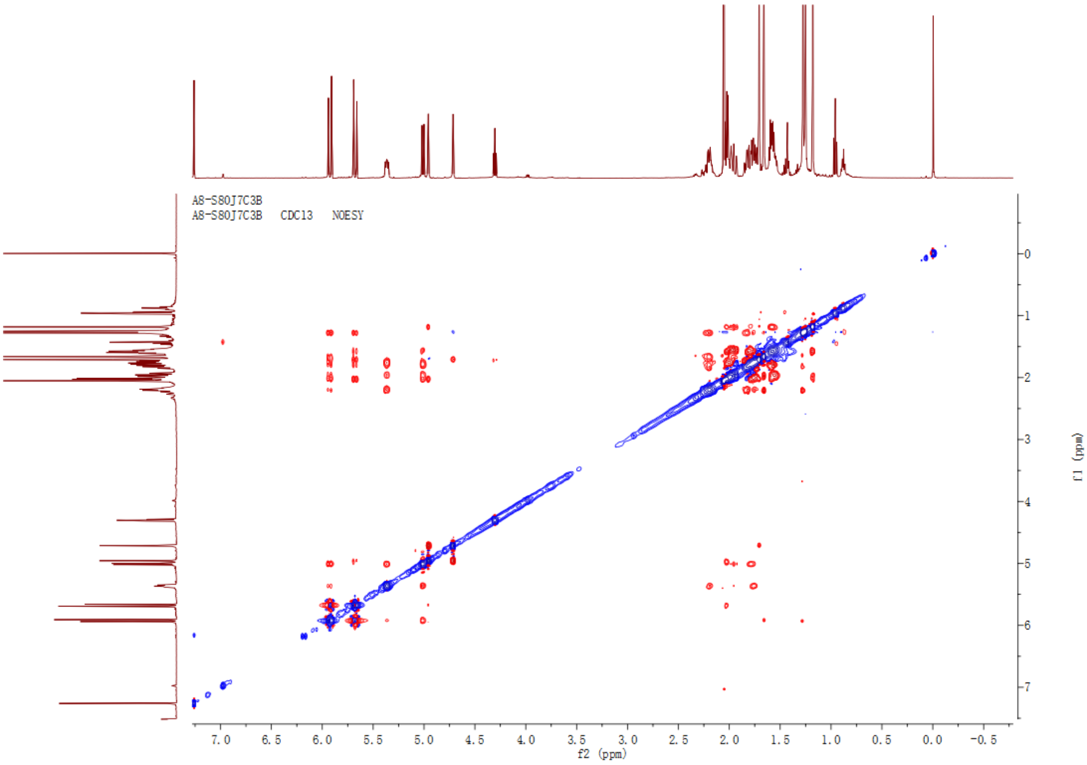

Figure S15. NOESY spectrum (500 MHz) of compound **2** in $CDCl_3$

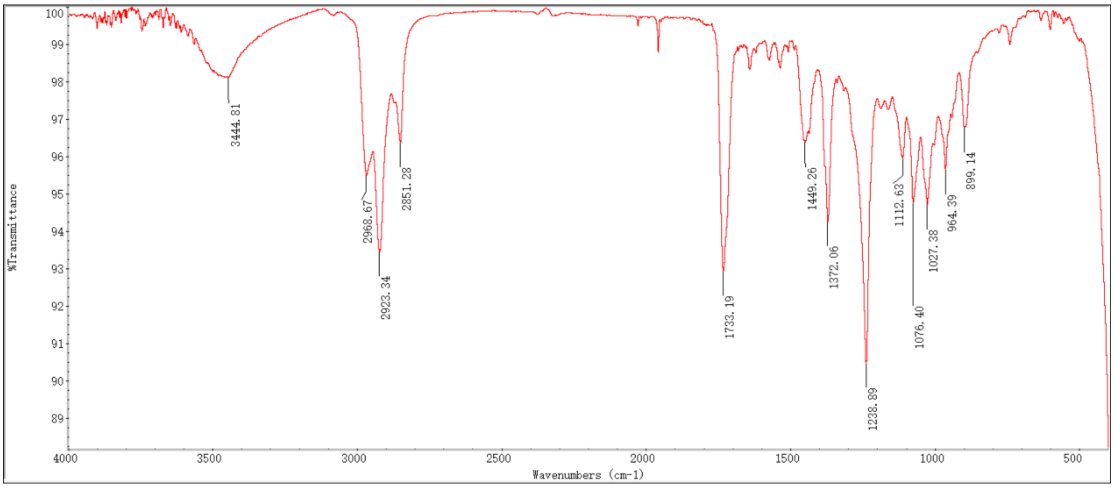

Figure S16. IR spectrum of compound **2**

LIST: h16289-c1 18-Aug-16 Elapse: 02:59.3 13
Samp: 14580G6C2O Start : 10:36:14 20
Comm: Finnigan/MAT95/70eV/R:10000
Mode: EI +VE +LMR BSCAN (EXP) UP HR NRM Study : S/N: PT200712-01-01
Oper: SIMM.CAS Client: S/N: PT001263 Inlet :
Limt: (0) . .
: (413) C22.H100.O3
Peak: 1000.00 mmu R+D: -2.0 > 60.0
Data: CMASS : converted

	26720			(mmu)		
Mass	Intensity	%RA	%RIC	Delta	R+D	Composition
80.06188 *	33043	14.22	0.24	0.7	3.0	C6.H8
81.07045 *	232438	100.00	1.70	0.0	2.5	C6.H9
82.07713 *	53934	23.20	0.39	1.1	2.0	C6.H10
83.08572 *	153134	65.88	1.12	0.4	1.5	C6.H11
84.09243 *	39935	17.18	0.29	1.5	1.0	C6.H12
85.10138 *	173031	74.44	1.26	0.3	0.5	C6.H13
91.05471 *	92804	39.93	0.68	0.1	4.5	C7.H7
93.07073 *	164504	70.77	1.20	-0.3	3.5	C7.H9
94.07835 *	53366	22.96	0.39	-0.1	3.0	C7.H10
95.08712 *	162230	69.79	1.18	-1.0	2.5	C7.H11
96.09487 *	37519	16.14	0.27	-1.0	2.0	C7.H12
97.06724 *	36240	15.59	0.26	-1.9	2.5	C6.H9.O
97.10329 *	144323	62.09	1.05	-1.6	1.5	C7.H13
99.11810 *	62461	26.87	0.46	-0.7	0.5	C7.H15
105.0326 *	93515	40.23	0.68	1.4	5.5	C7.H5.O
106.0357 *	36738	15.81	0.27			
107.0411 *	135014	58.09	0.99			
108.0466 *	49955	21.49	0.36			
109.0540 *	93088	40.05	0.68			
110.0614 *	27429	11.80	0.20			
111.0718 *	107229	46.13	0.78			
112.0811 *	27002	11.62	0.20			
113.0931 *	50381	21.68	0.37			
119.0857 *	155266	66.80	1.13	0.4	4.5	C9.H11
120.0926 *	56563	24.33	0.41	1.3	4.0	C9.H12
121.1015 *	132598	57.05	0.97	0.2	3.5	C9.H13
122.1086 *	48534	20.88	0.35	1.0	3.0	C9.H14
123.0808 *	68217	29.35	0.50	0.2	3.5	C8.H11.O
123.1170 *	64451	27.73	0.47	0.3	2.5	C9.H15
125.0965 *	81932	35.25	0.60	0.2	2.5	C8.H13.O
125.1323 *	56208	24.18	0.41	0.8	1.5	C9.H17
127.1487 *	34322	14.77	0.25	-0.1	0.5	C9.H19
131.0863 *	31479	13.54	0.23	-0.2	5.5	C10.H11
132.0935 *	39651	17.06	0.29	0.4	5.0	C10.H12
133.1017 *	98276	42.28	0.72	0.0	4.5	C10.H13
134.1088 *	124639	53.62	0.91	0.8	4.0	C10.H14
135.0807 *	70847	30.48	0.52	0.3	4.5	C9.H11.O
135.1168 *	72907	31.37	0.53	0.6	3.5	C10.H15
136.0875 *	32971	14.18	0.24	1.3	4.0	C9.H12.O
137.1330 *	30484	13.11	0.22	0.1	2.5	C10.H17
145.1017 *	50879	21.89	0.37	0.0	5.5	C11.H13
147.1171 *	56137	24.15	0.41	0.3	4.5	C11.H15
148.0885 *	47255	20.33	0.34	0.3	5.0	C10.H12.O
148.1245 *	35103	15.10	0.26	0.7	4.0	C11.H16
149.0244 *	84064	36.17	0.61	-0.6	6.5	C8.H5.O3
149.0967 *	28210	12.14	0.21	-0.1	4.5	C10.H13.O
149.1326 *	40575	17.46	0.30	0.4	3.5	C11.H17
150.1397 *	28139	12.11	0.21	1.1	3.0	C11.H18
151.1117 *	47041	20.24	0.34	0.5	3.5	C10.H15.O
153.0922 *	34890	15.01	0.25	-0.6	3.5	C9.H13.O2
159.1173 *	55284	23.78	0.40	0.0	5.5	C12.H15
161.1323 *	37377	16.08	0.27	0.8	4.5	C12.H17
173.1325 *	33611	14.46	0.25	0.6	5.5	C13.H17
187.1476 *	30271	13.02	0.22	1.0	5.5	C14.H19
286.2298 *	28139	12.11	0.21	-0.1	6.0	C20.H30.O
346.2507 *	70491	30.33	0.51	0.1	6.0	C22.H34.O3

Figure S17. HREIMS spectrum of compound **3**

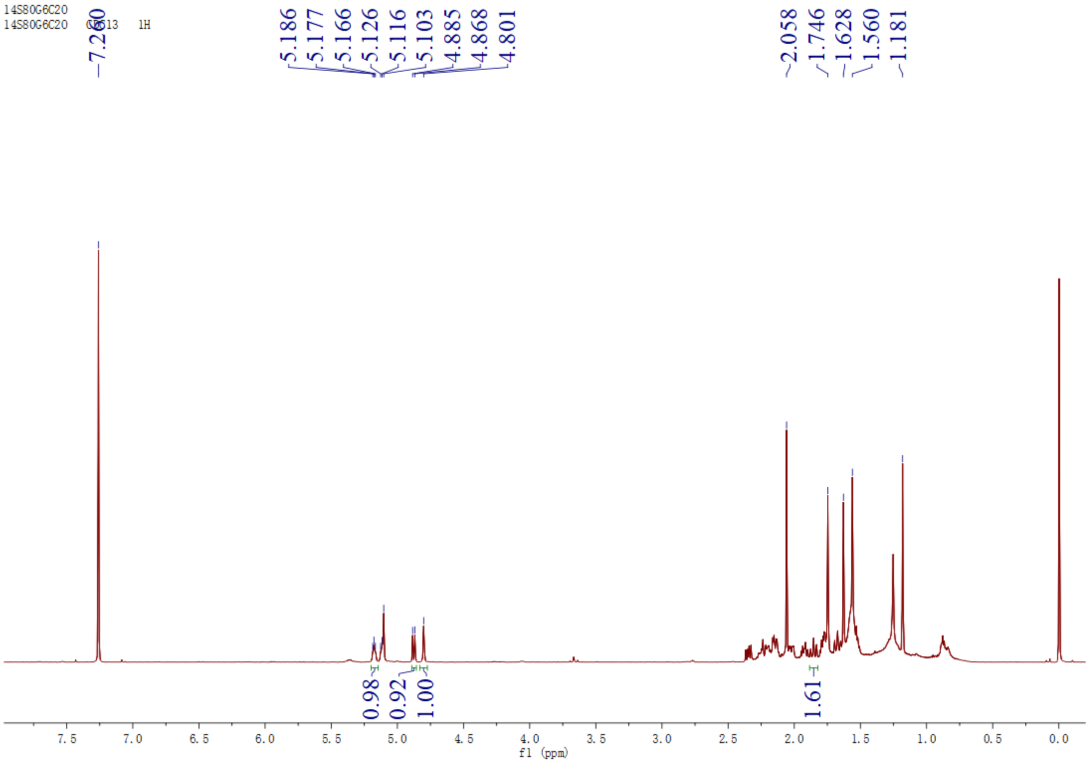

Figure S18. ^{1}H NMR spectrum (600 MHz) of compound **3** in $CDCl_3$

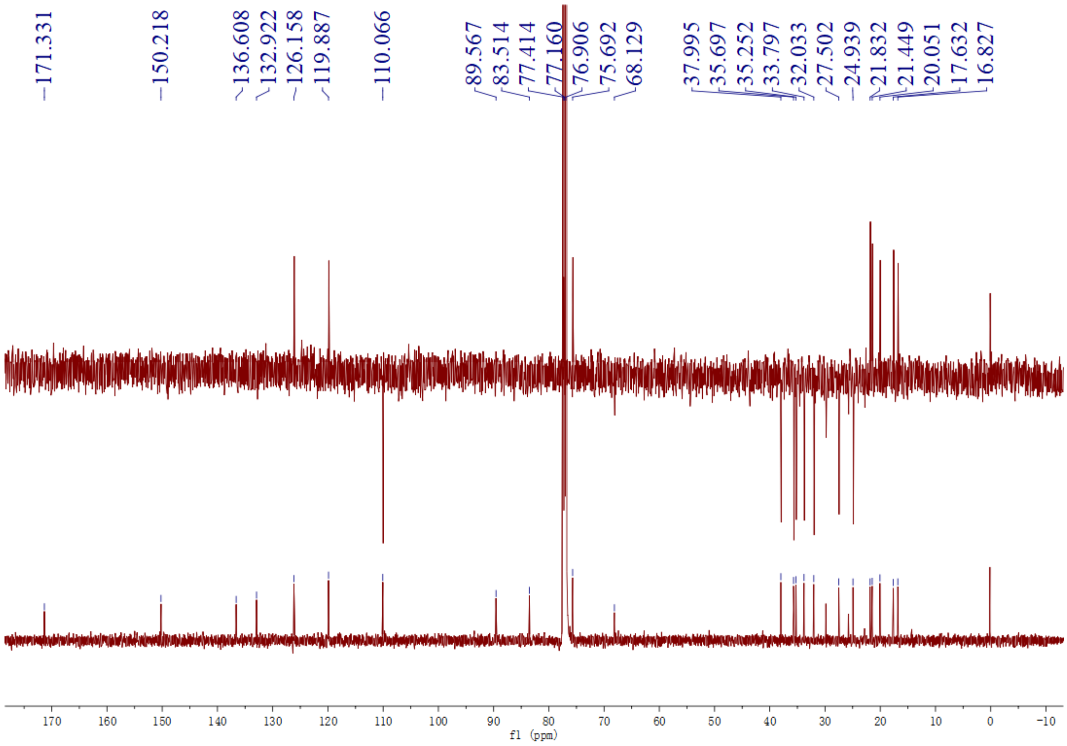

Figure S19. ^{13}C NMR (BB+DEPT) spectrum (125 MHz) of compound **3** in $CDCl_3$

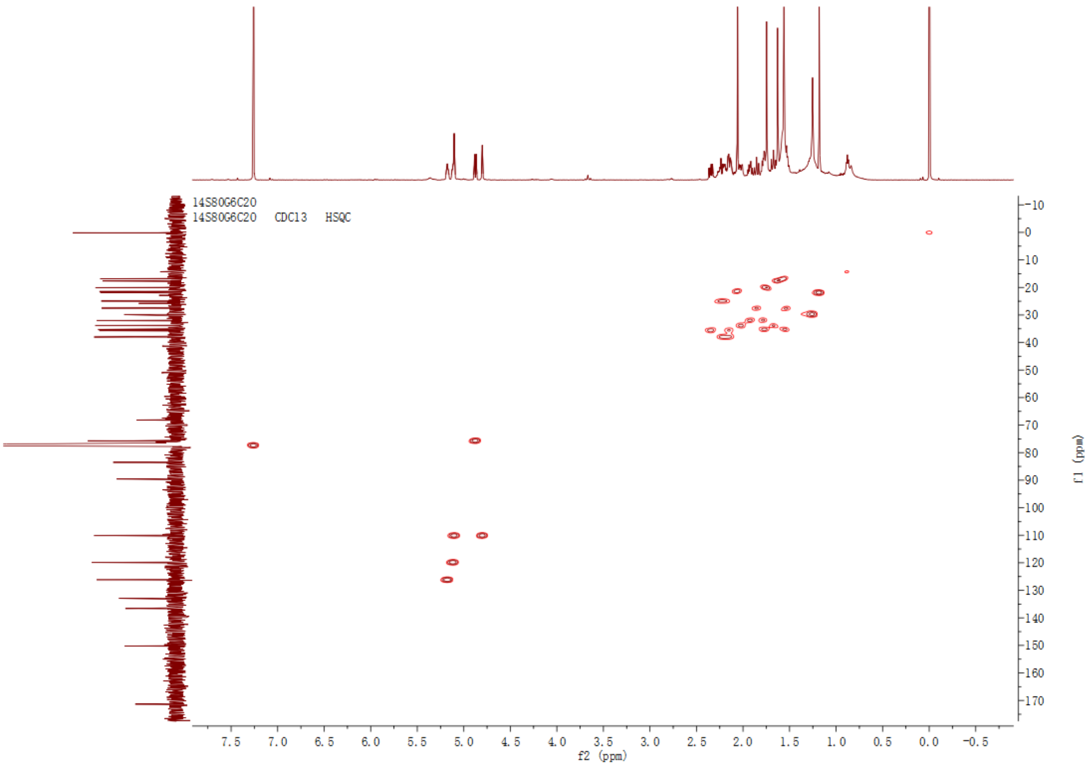

Figure S20. HSQC spectrum (600 MHz) of compound **3** in $CDCl_3$

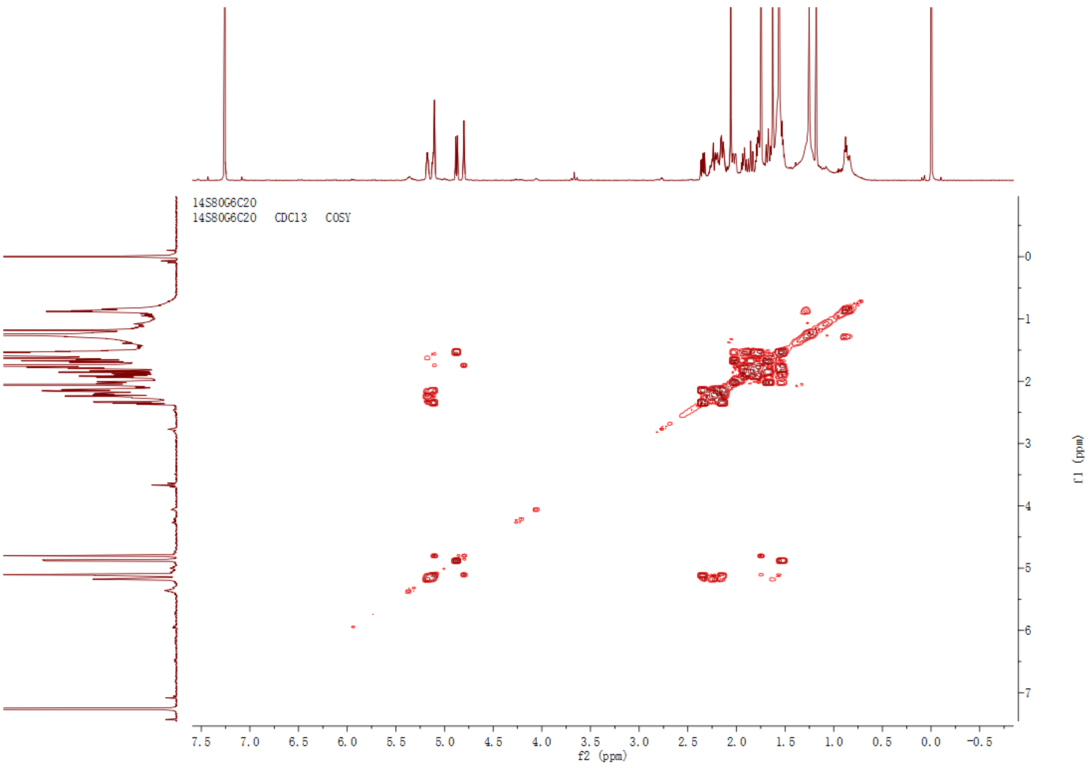

Figure S21. ^{1}H–^{1}H COSY spectrum (600 MHz) of compound **3** in $CDCl_3$

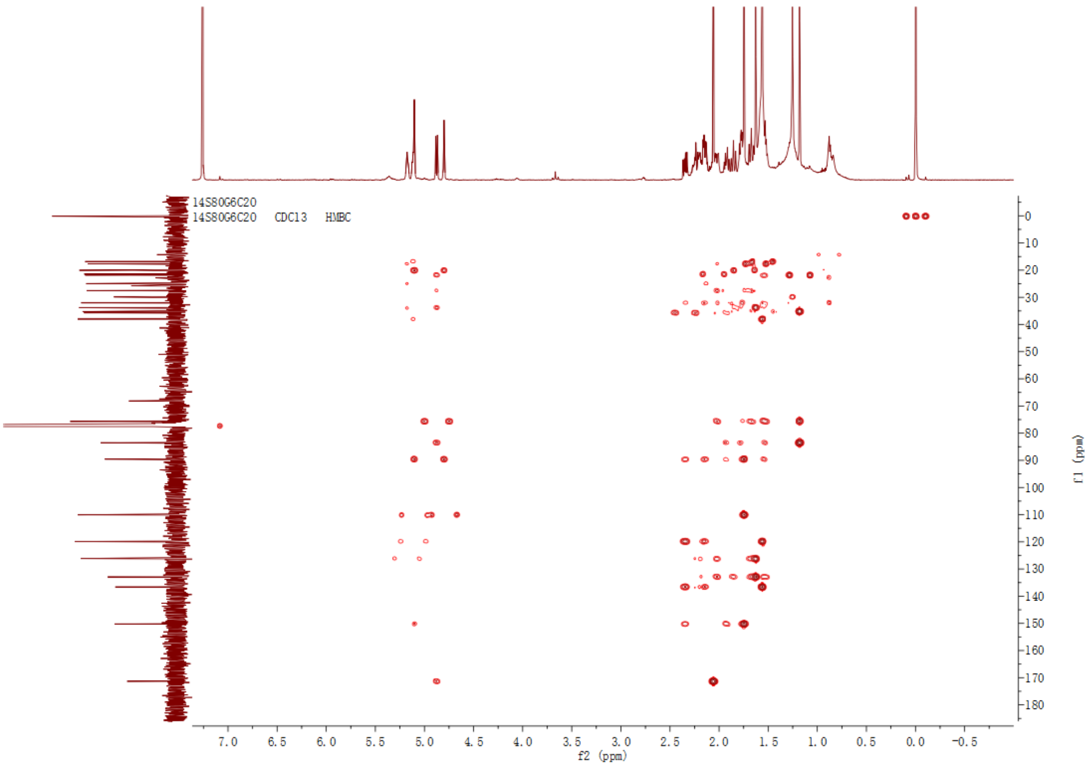

Figure S22. HMBC spectrum (600 MHz) of compound **3** in $CDCl_3$

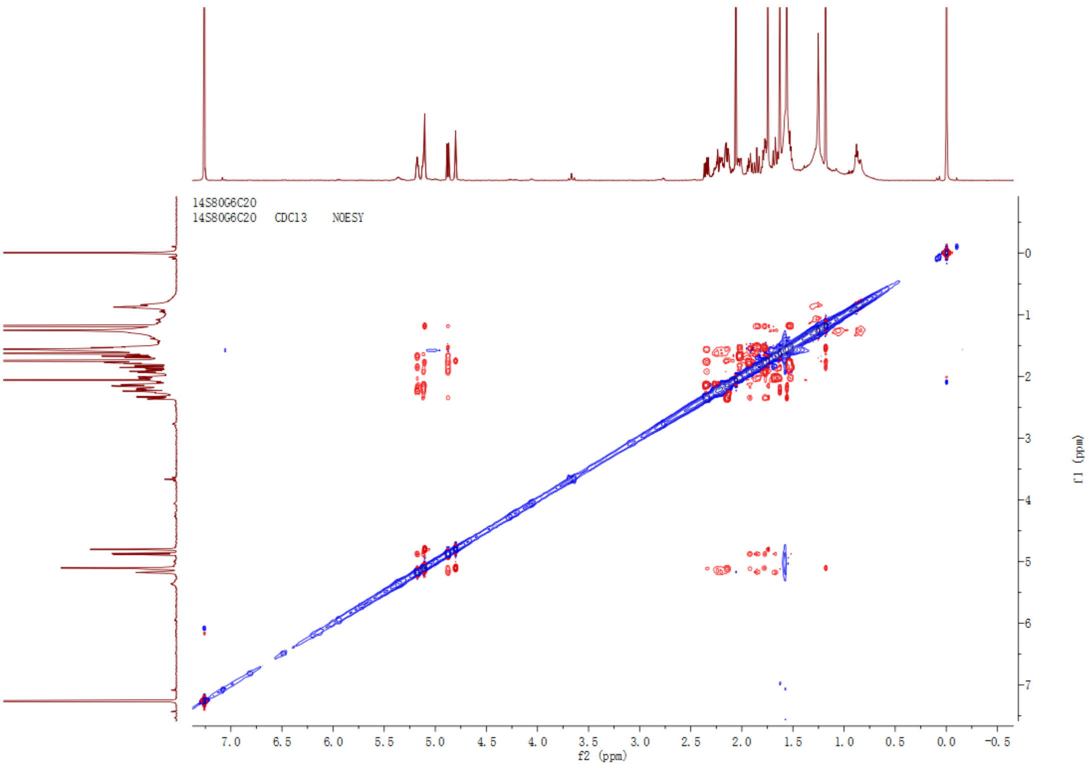

Figure S23. NOESY spectrum (600 MHz) of compound **3** in $CDCl_3$

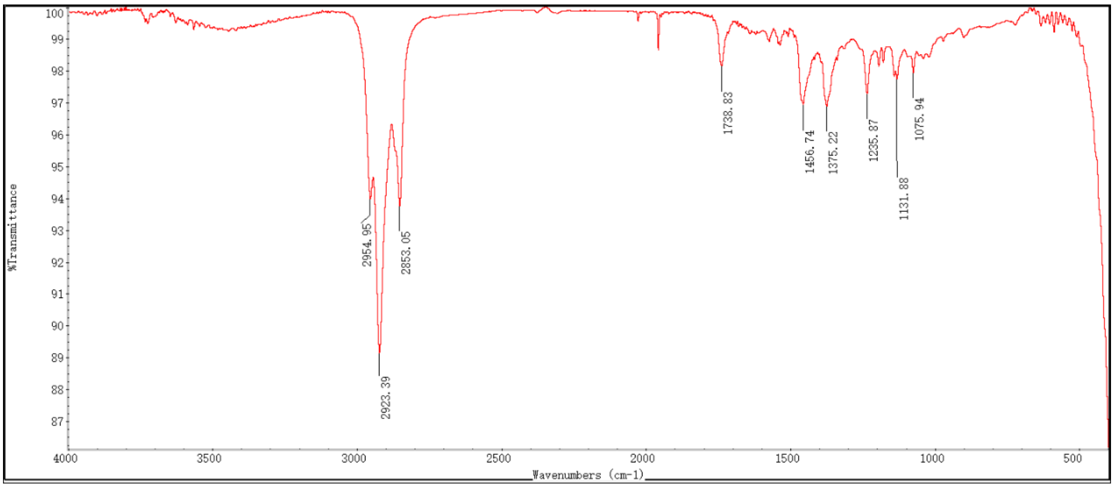

Figure S24. IR spectrum of compound **3**

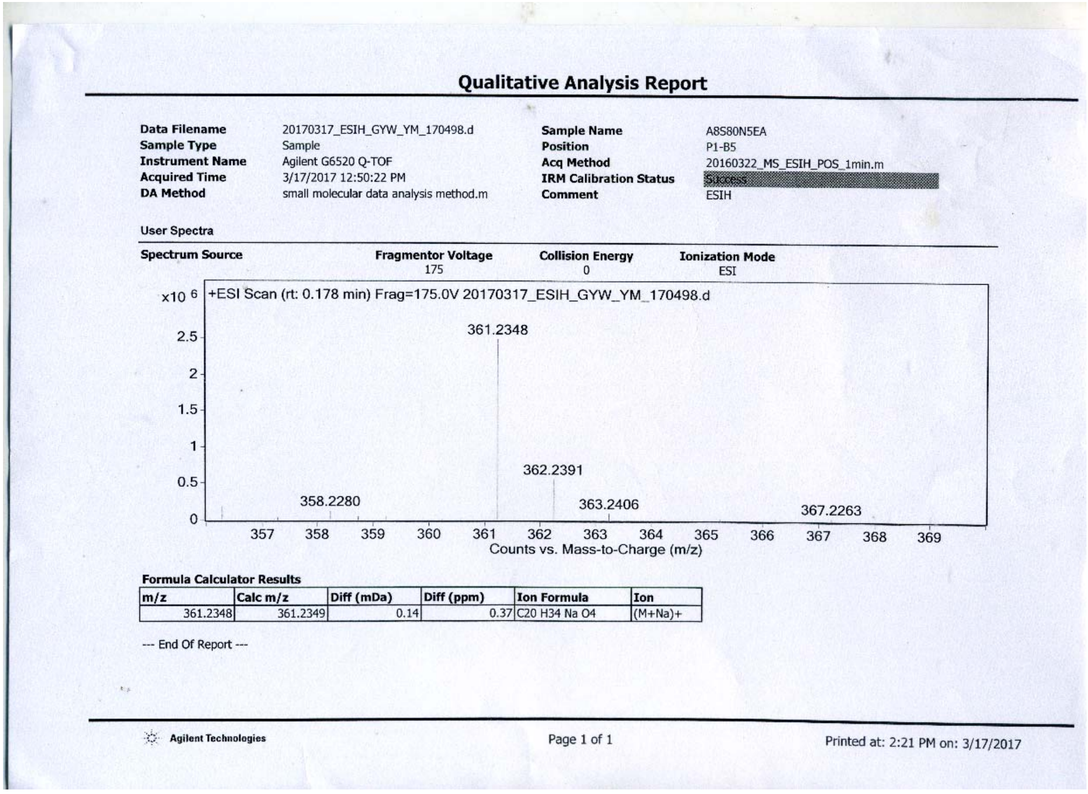

Qualitative Analysis Report

**Data Filename**	20170317_ESIH_GYW_YM_170498.d	**Sample Name**	A8S80N5EA
**Sample Type**	Sample	**Position**	P1-B5
**Instrument Name**	Agilent G6520 Q-TOF	**Acq Method**	20160322_MS_ESIH_POS_1min.m
**Acquired Time**	3/17/2017 12:50:22 PM	**IRM Calibration Status**	Success
**DA Method**	small molecular data analysis method.m	**Comment**	ESIH

User Spectra

**Spectrum Source**	**Fragmentor Voltage**	**Collision Energy**	**Ionization Mode**
	175	0	ESI

Formula Calculator Results

m/z	Calc m/z	Diff (mDa)	Diff (ppm)	Ion Formula	Ion
361.2348	361.2349	0.14	0.37	C20 H34 Na O4	(M+Na)+

--- End Of Report ---

Agilent Technologies Page 1 of 1 Printed at: 2:21 PM on: 3/17/2017

Figure S25. HRESIMS spectrum of compound **4**

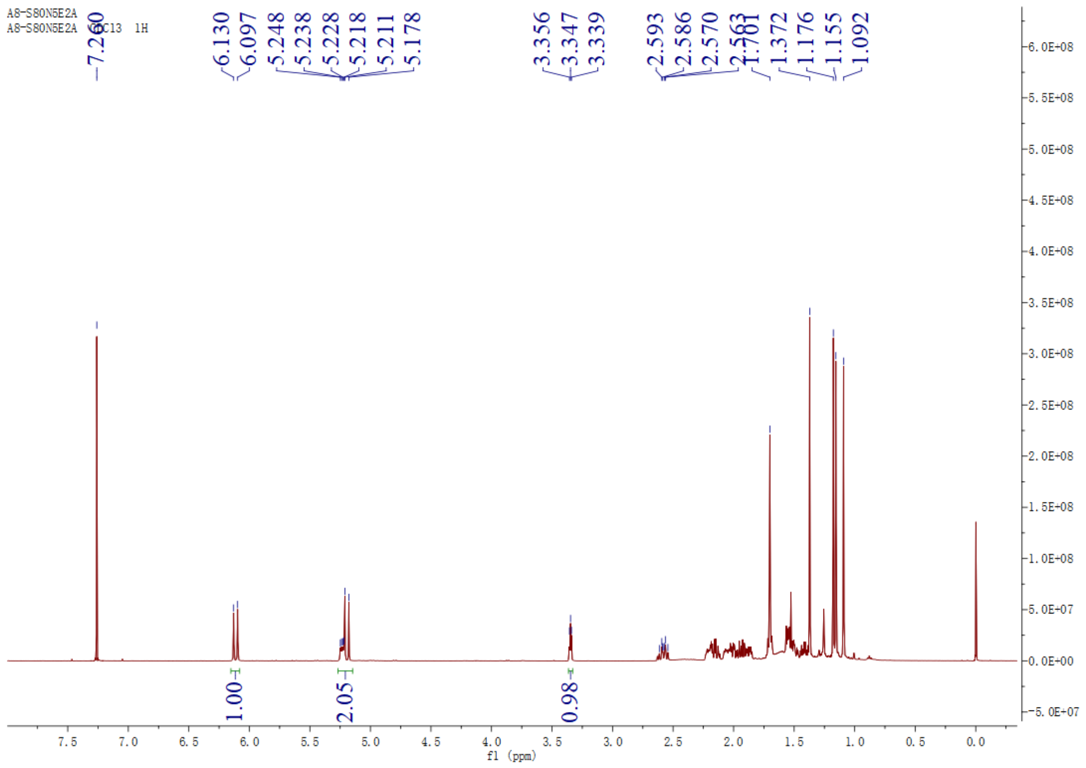

Figure S26. ^{1}H NMR spectrum (500 MHz) of compound **4** in $CDCl_3$

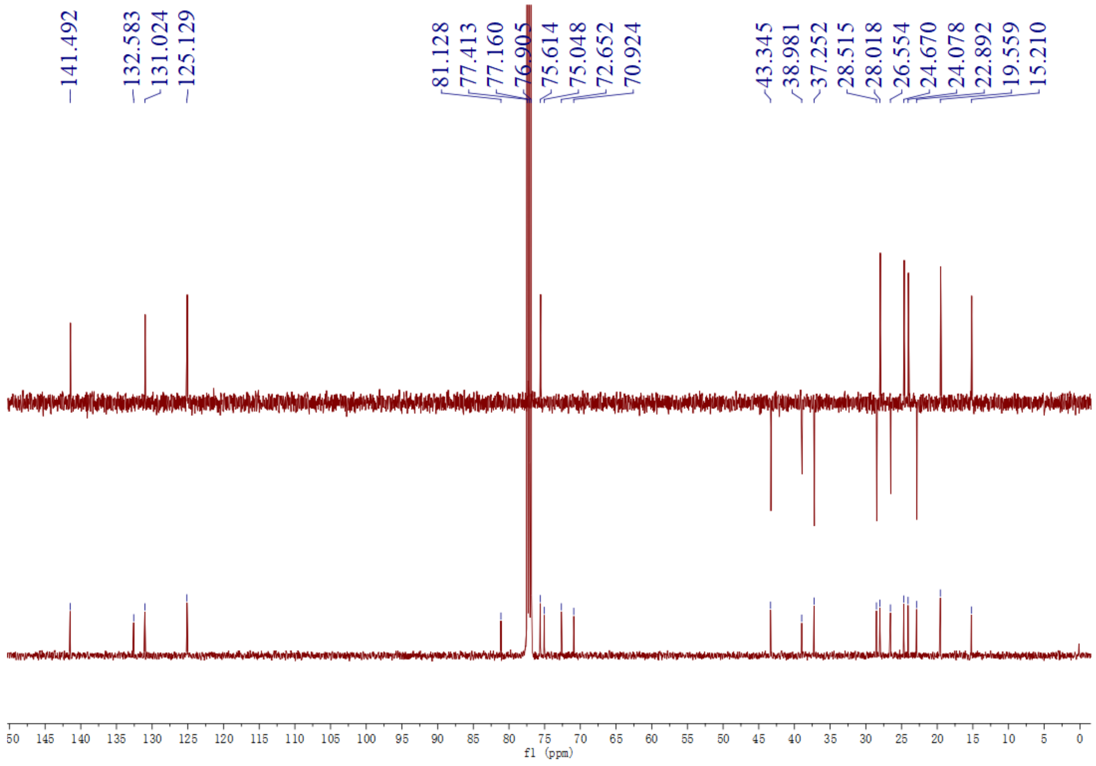

Figure S27. ^{13}C NMR (BB+DEPT) spectrum (125 MHz) of compound **4** in $CDCl_3$

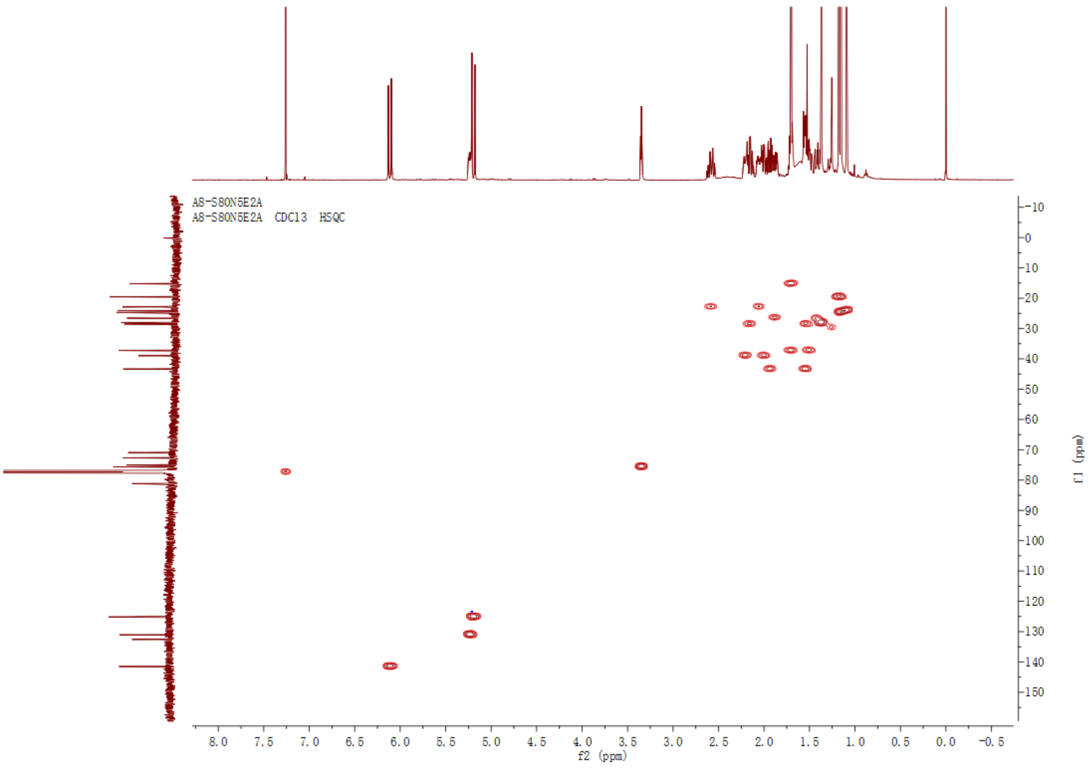

Figure S28. HSQC spectrum (500 MHz) of compound **4** in $CDCl_3$

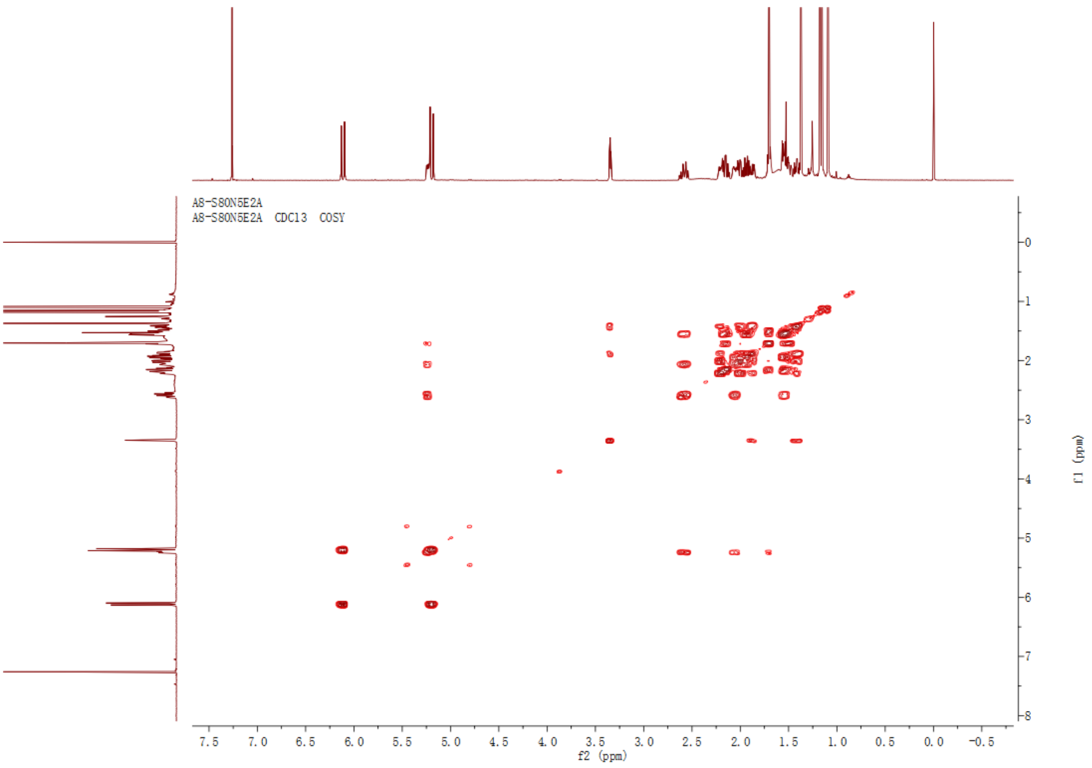

Figure S29. ^{1}H–^{1}H COSY spectrum (500 MHz) of compound **4** in $CDCl_3$

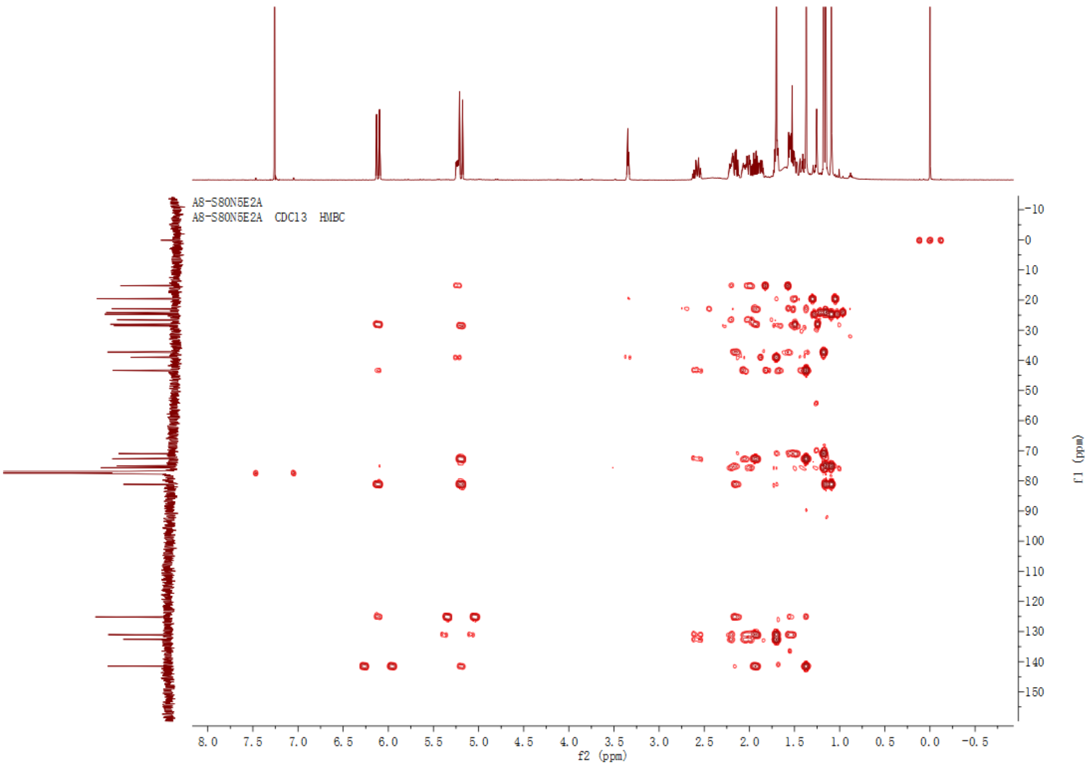

Figure S30. HMBC spectrum (500 MHz) of compound **4** in $CDCl_3$

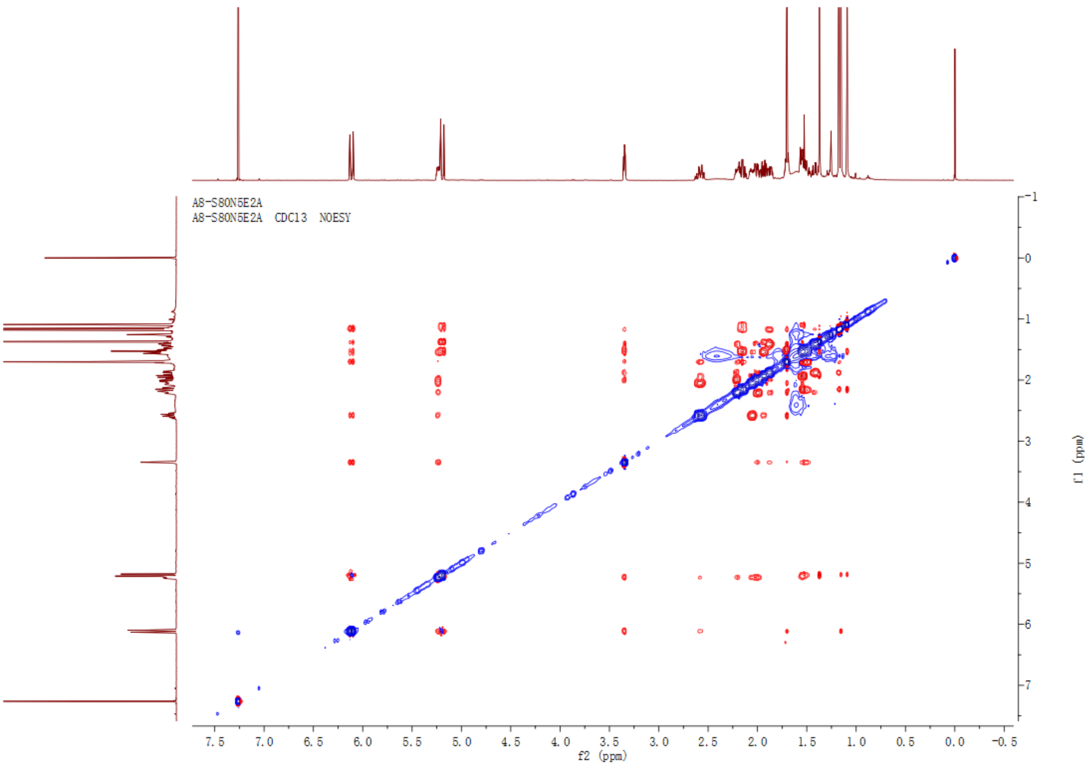

Figure S31. NOESY spectrum (500 MHz) of compound **4** in $CDCl_3$

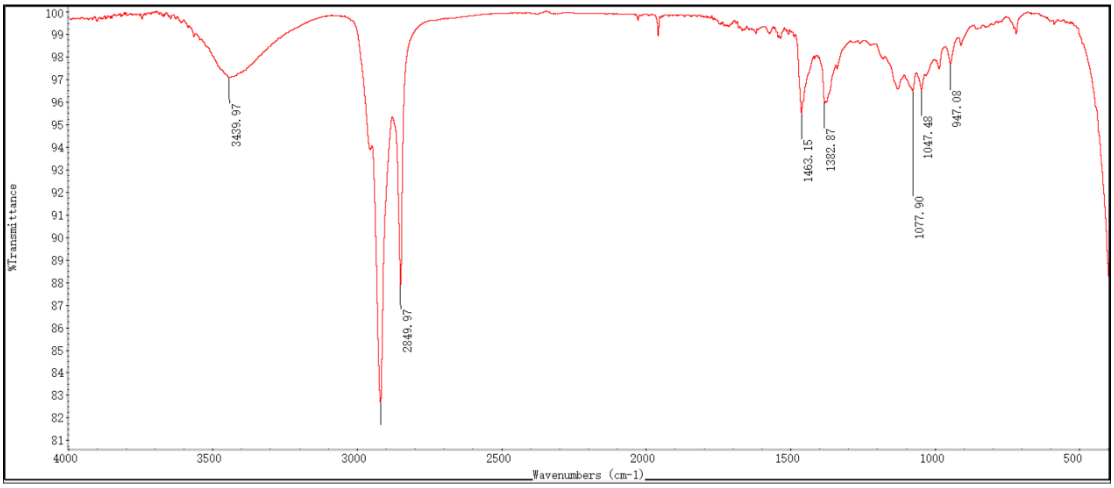

Figure S32. IR spectrum of compound **4**

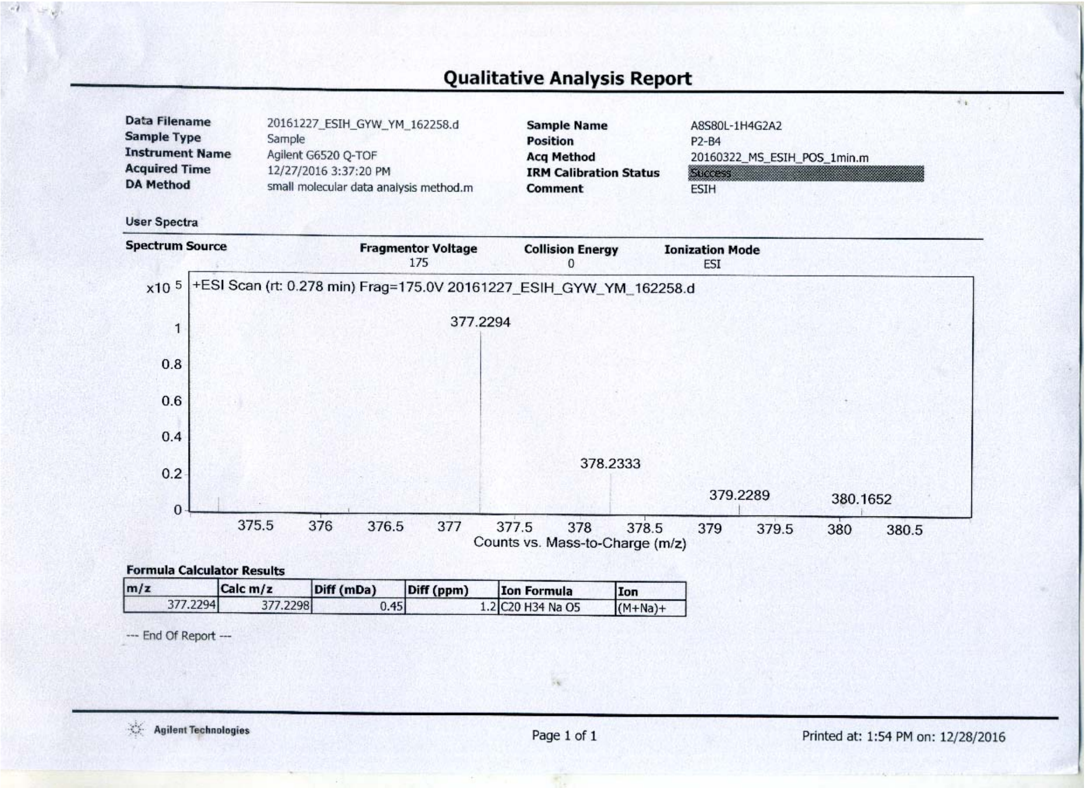

Qualitative Analysis Report

**Data Filename**	20161227_ESIH_GYW_YM_162258.d	**Sample Name**	A8S80L-1H4G2A2
**Sample Type**	Sample	**Position**	P2-B4
**Instrument Name**	Agilent G6520 Q-TOF	**Acq Method**	20160322_MS_ESIH_POS_1min.m
**Acquired Time**	12/27/2016 3:37:20 PM	**IRM Calibration Status**	Success
**DA Method**	small molecular data analysis method.m	**Comment**	ESIH

User Spectra

Spectrum Source	Fragmentor Voltage	Collision Energy	Ionization Mode
	175	0	ESI

Formula Calculator Results

m/z	Calc m/z	Diff (mDa)	Diff (ppm)	Ion Formula	Ion
377.2294	377.2298	0.45	1.2	C20 H34 Na O5	(M+Na)+

--- End Of Report ---

Agilent Technologies Page 1 of 1 Printed at: 1:54 PM on: 12/28/2016

Figure S33. HRESIMS spectrum of compound **5**

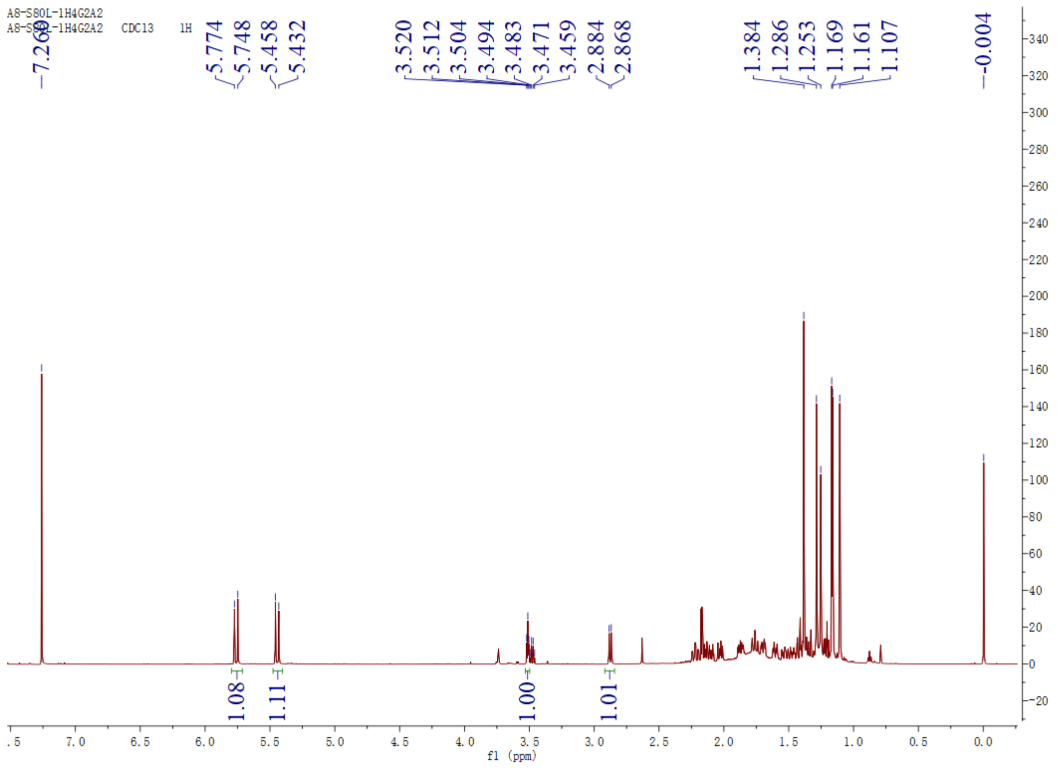

Figure S34. ^{1}H NMR spectrum (600 MHz) of compound **5** in $CDCl_3$.

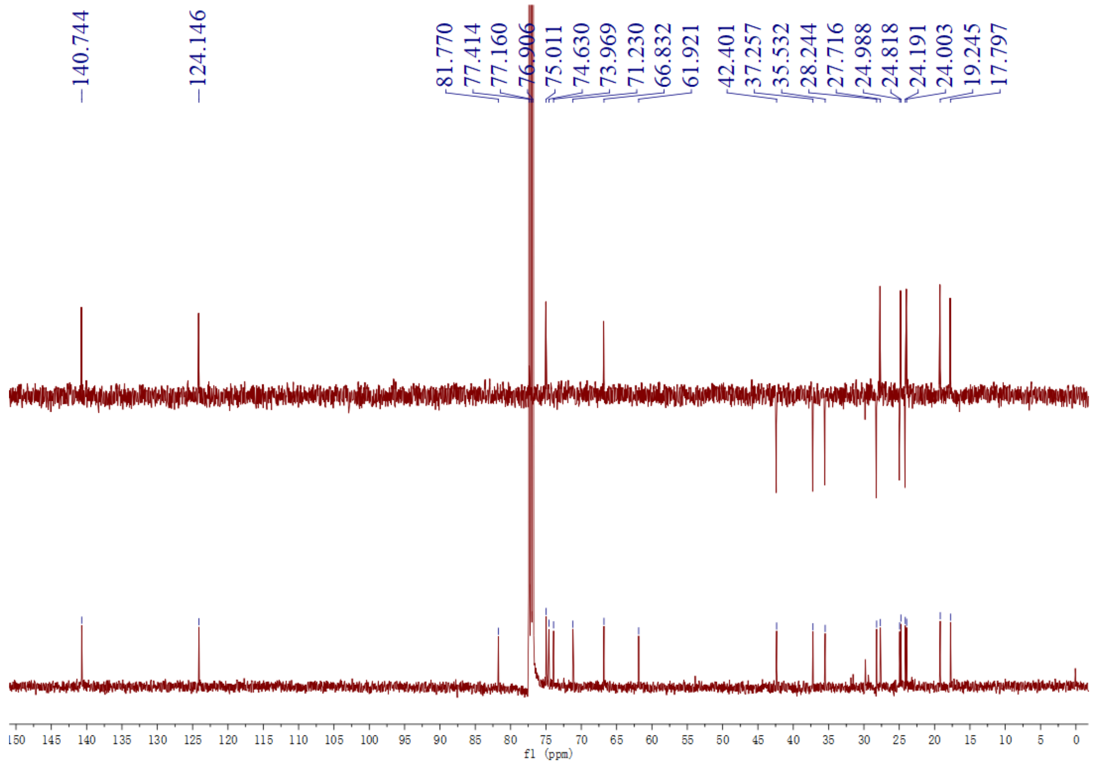

Figure S35. ^{13}C NMR (BB+DEPT) spectrum (125 MHz) of compound **5** in $CDCl_3$

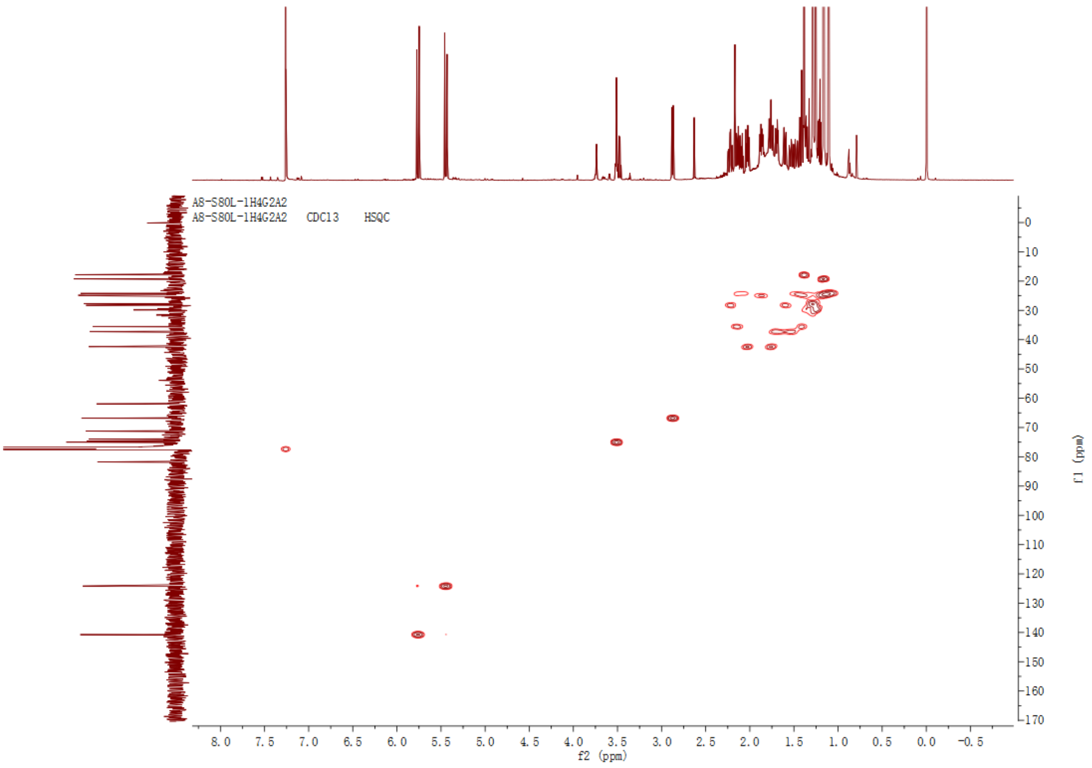

Figure S36. HSQC spectrum (600 MHz) of compound **5** in $CDCl_3$

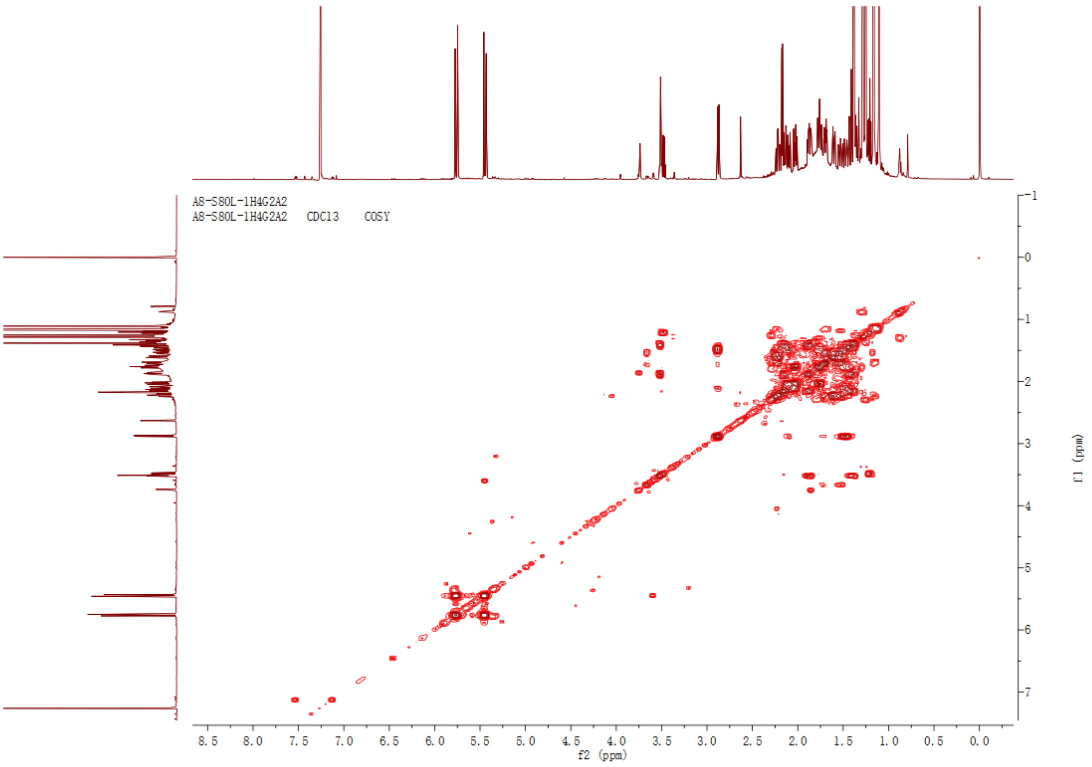

Figure S37. ^1H–^1H COSY spectrum (600 MHz) of compound **5** in $CDCl_3$

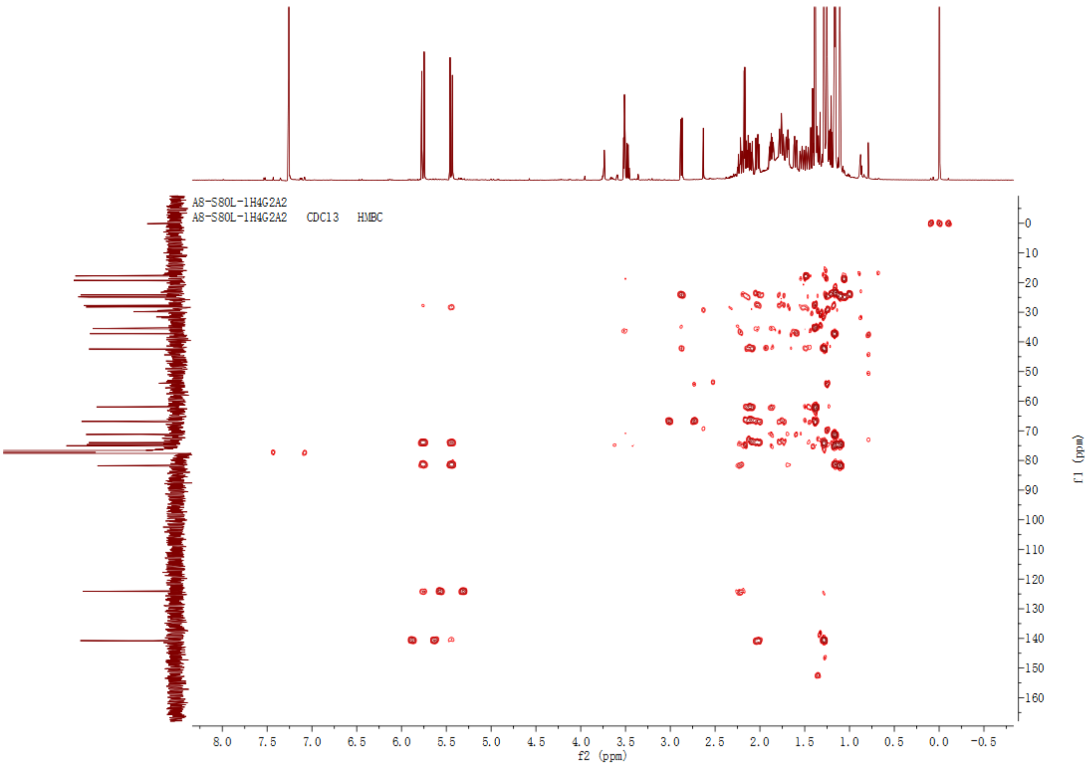

Figure S38. HMBC spectrum (600 MHz) of compound **5** in $CDCl_3$

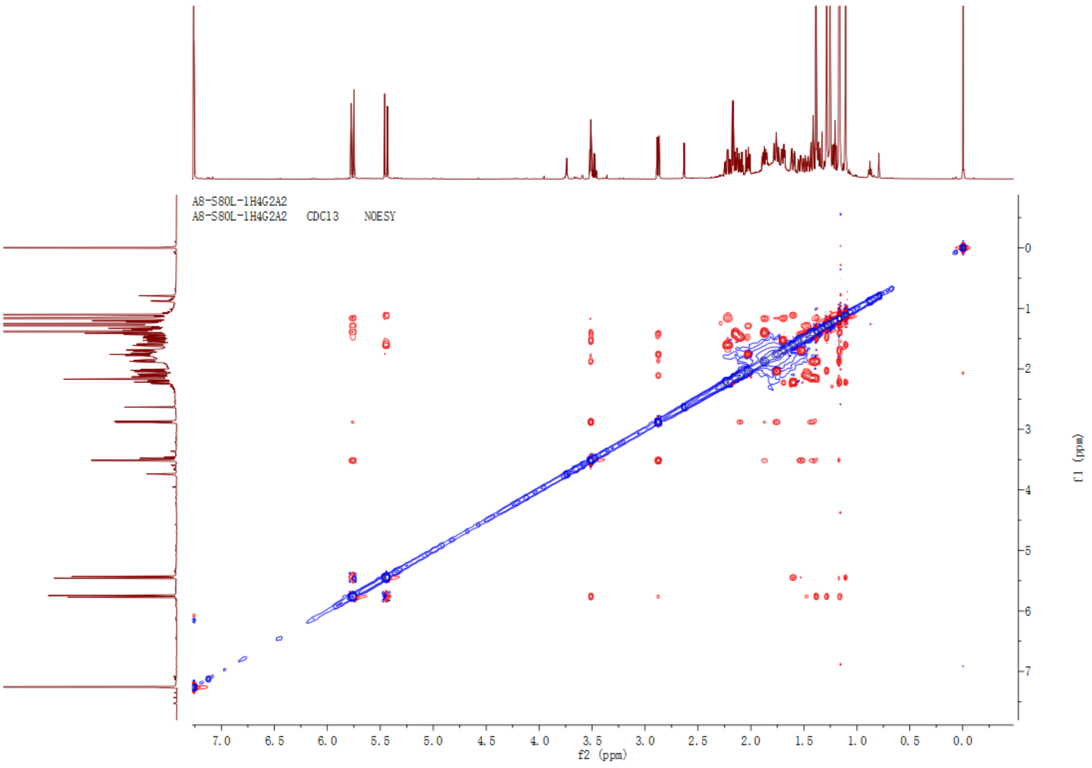

Figure S39. NOESY spectrum (600 MHz) of compound **5** in $CDCl_3$

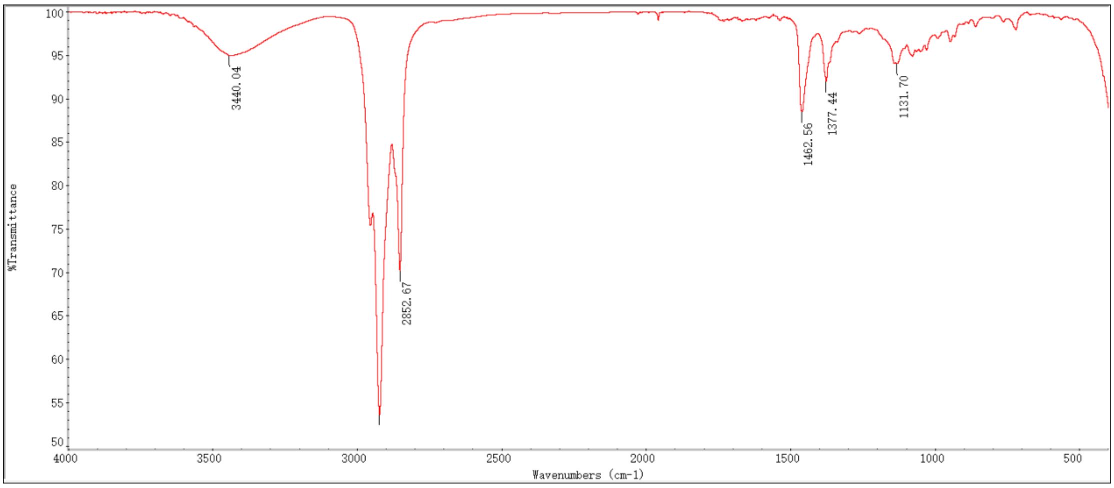

Figure S40. IR spectrum of compound **5**

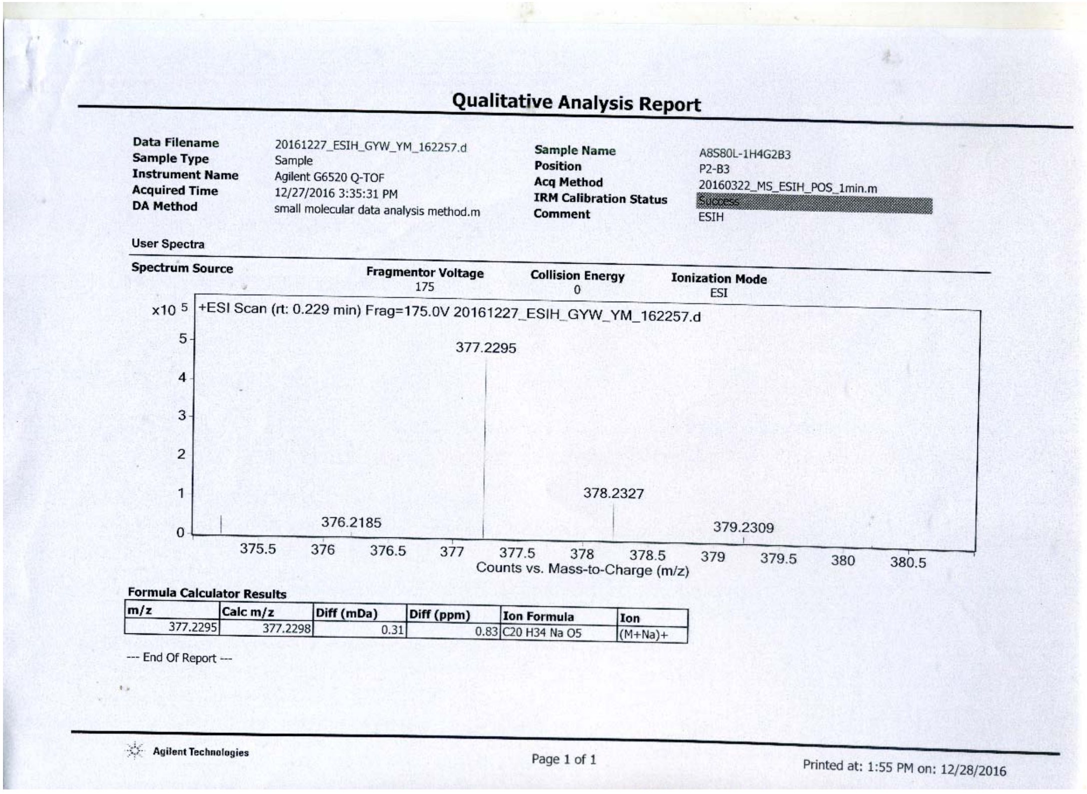

Qualitative Analysis Report

**Data Filename**	20161227_ESIH_GYW_YM_162257.d	**Sample Name**	A8S80L-1H4G2B3
**Sample Type**	Sample	**Position**	P2-B3
**Instrument Name**	Agilent G6520 Q-TOF	**Acq Method**	20160322_MS_ESIH_POS_1min.m
**Acquired Time**	12/27/2016 3:35:31 PM	**IRM Calibration Status**	Success
**DA Method**	small molecular data analysis method.m	**Comment**	ESIH

User Spectra

Spectrum Source	Fragmentor Voltage	Collision Energy	Ionization Mode
	175	0	ESI

Formula Calculator Results

m/z	Calc m/z	Diff (mDa)	Diff (ppm)	Ion Formula	Ion
377.2295	377.2298	0.31	0.83	C20 H34 Na O5	(M+Na)+

--- End Of Report ---

Agilent Technologies | Page 1 of 1 | Printed at: 1:55 PM on: 12/28/2016

Figure S41. HRESIMS spectrum of compound **6**

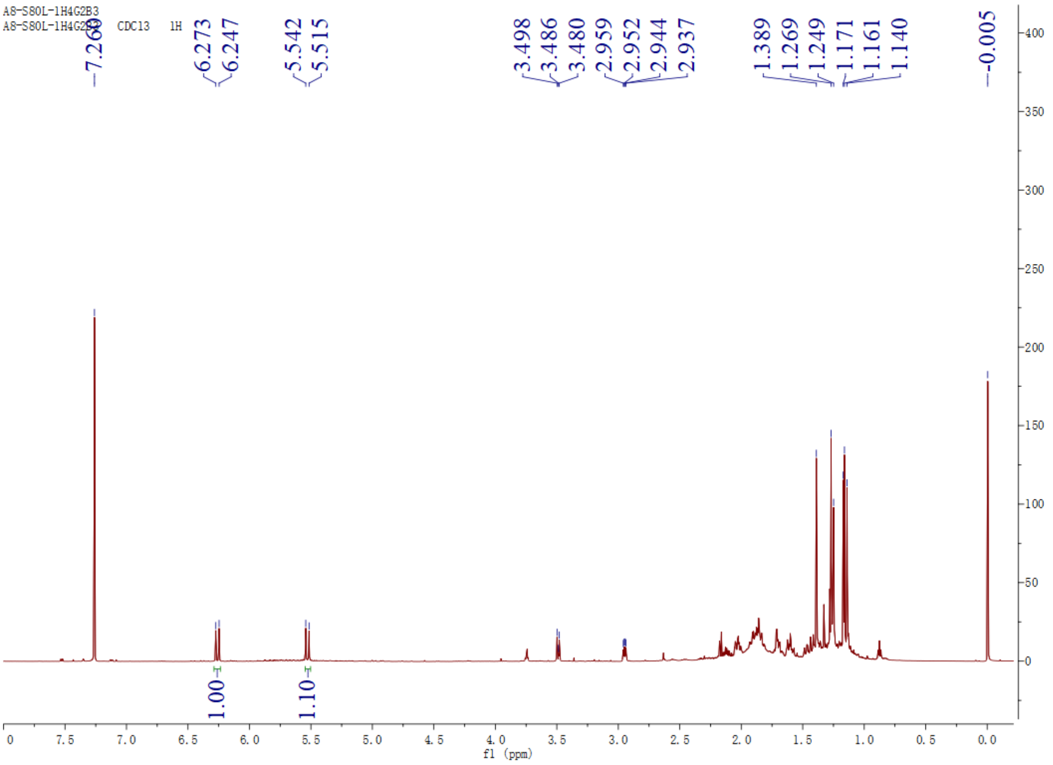

Figure S42. ^{1}H NMR spectrum (600 MHz) of compound **6** in $CDCl_3$

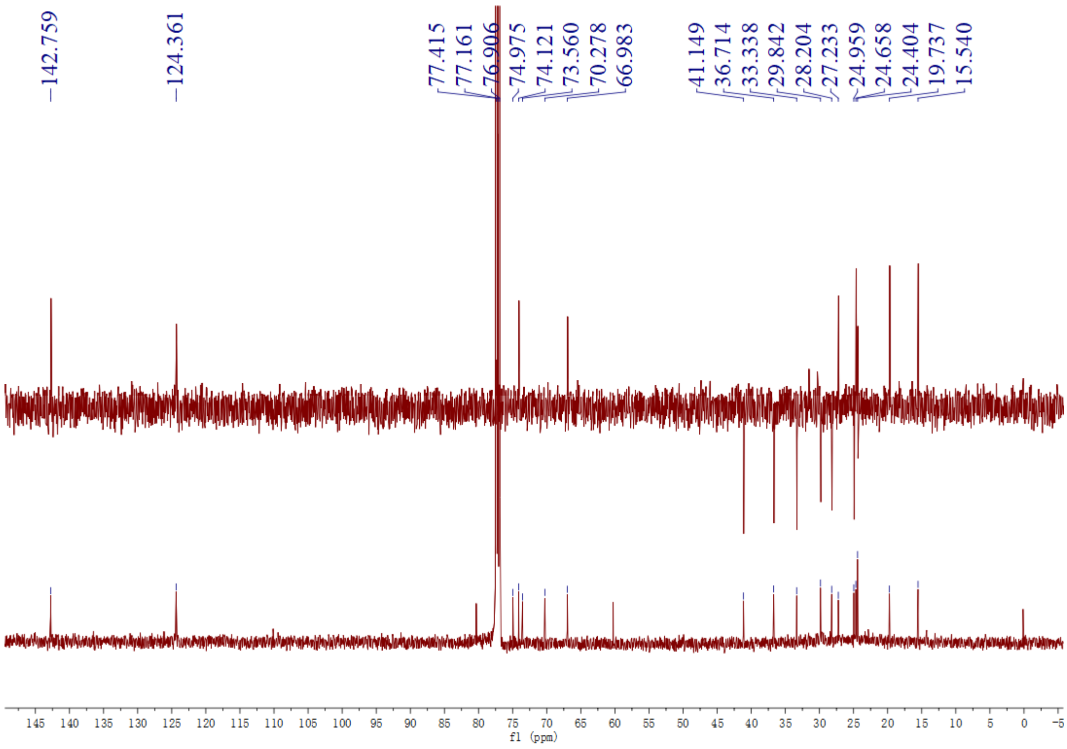

Figure S43. ^{13}C NMR (BB+DEPT) spectrum (125 MHz) of compound **6** in $CDCl_3$

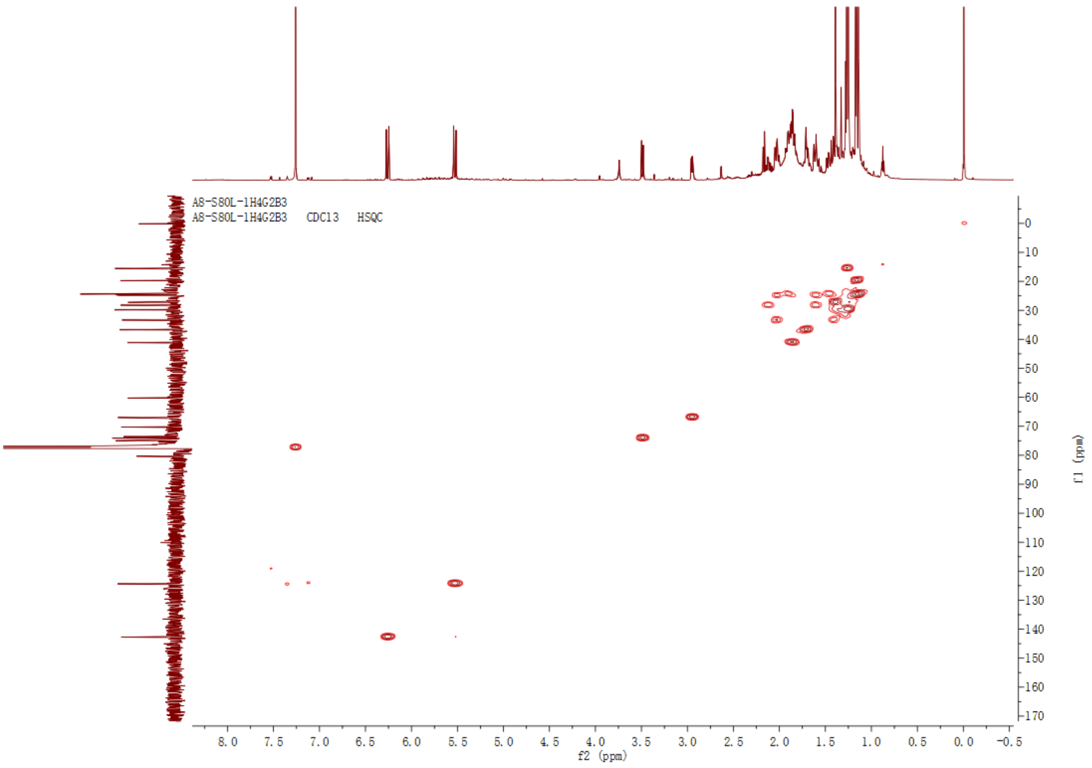

Figure S44. HSQC spectrum (600 MHz) of compound **6** in $CDCl_3$

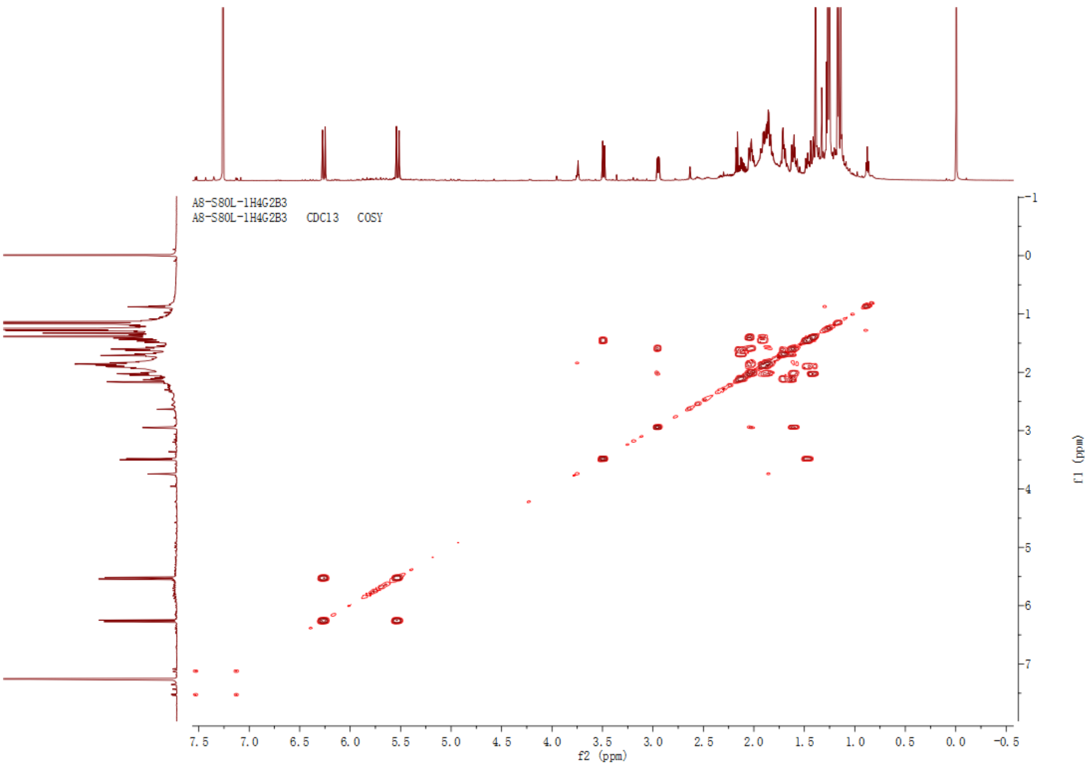

Figure S45. ^{1}H–^{1}H COSY spectrum (600 MHz) of compound **6** in $CDCl_3$

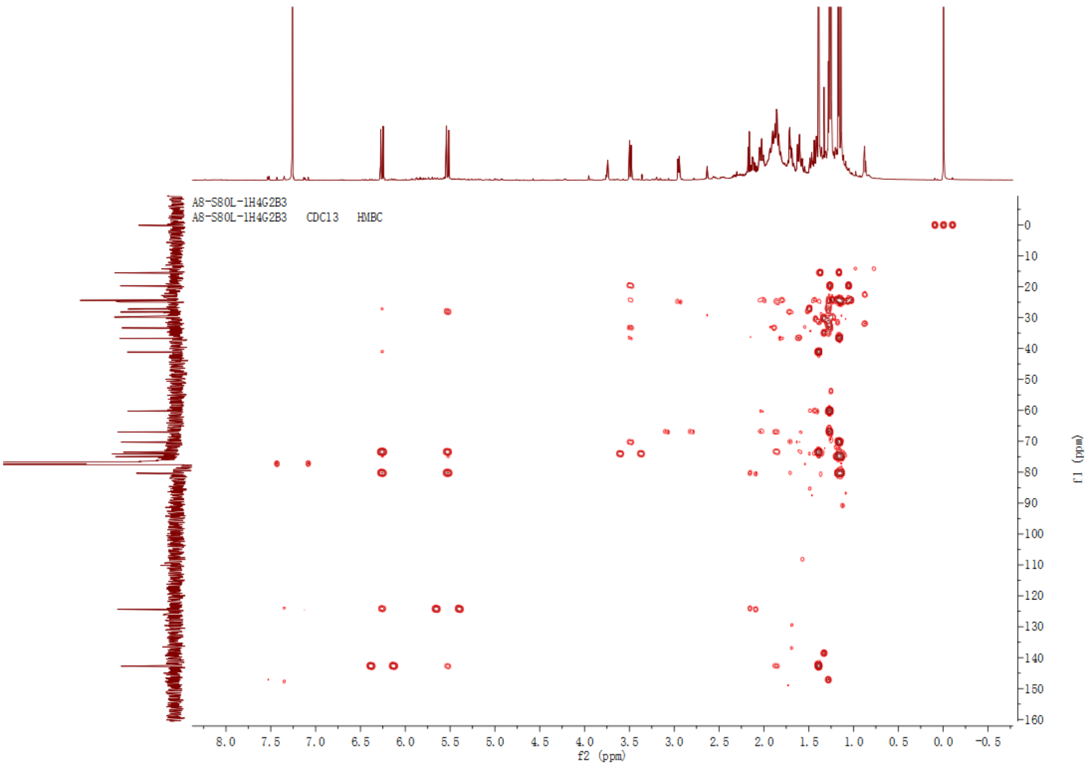

Figure S46. HMBC spectrum (600 MHz) of compound **6** in $CDCl_3$

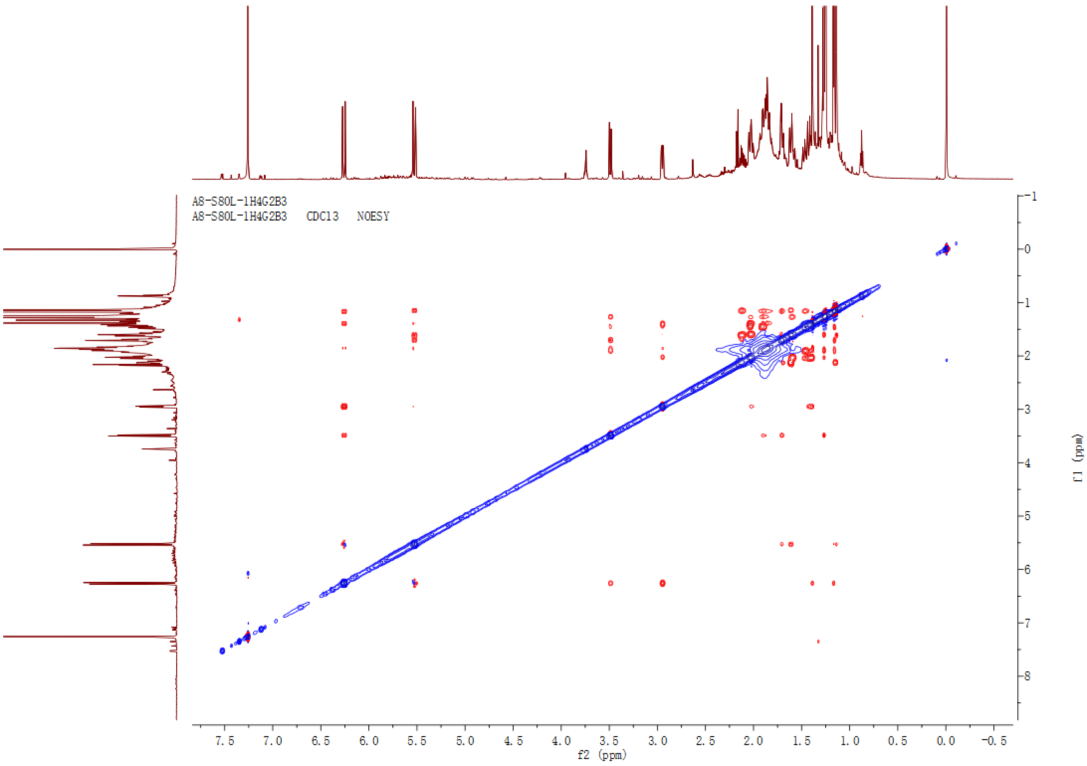

Figure S47. NOESY spectrum (600 MHz) of compound **6** in $CDCl_3$

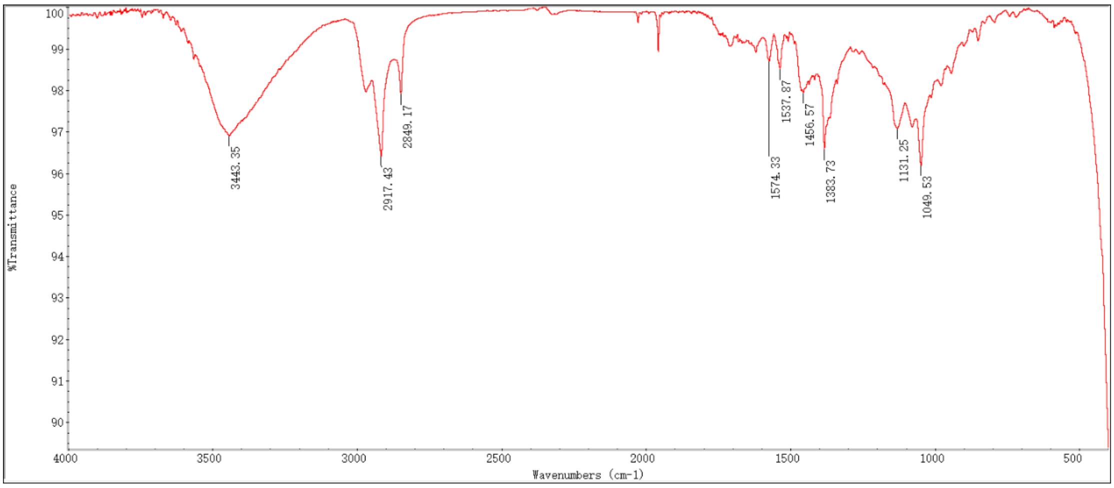

Figure S48. IR spectrum of compound **6**

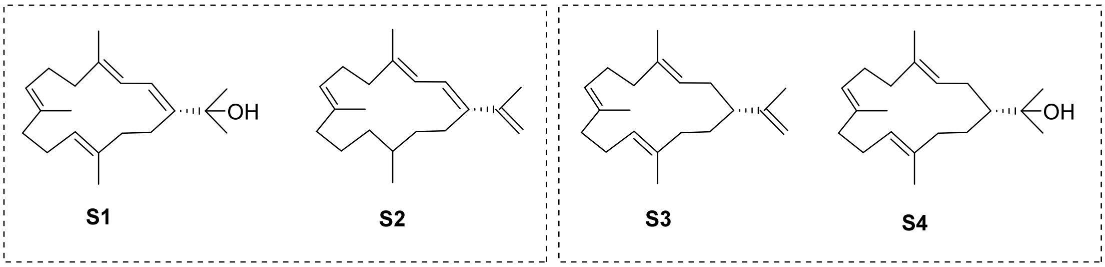

Sarcophyton crassocaule [1]   *Nephthea brassica* [2]

Figure S49. Examples of the coexistence of dehydration derivatives and their related alcohol precursors from different soft corals. [1,2]

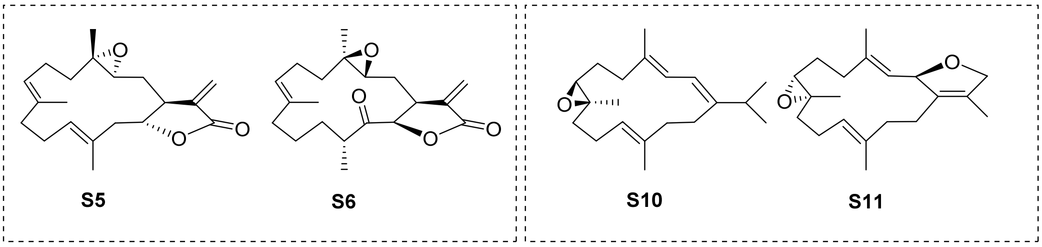

Sarcophyton crassocaule [3]   *Sarcophyton ehrenbergi* [5]

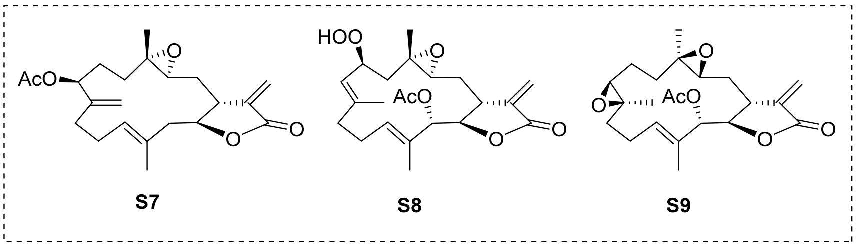

Sarcophyton crassocaule [4]

Figure S50. Examples of the coexistence of epimeric epoxy analogues from different soft corals. [3–5]

References

1. Bowden, B.F.; Coll, J.C.; Mitchell, S.J. Studies of Australian soft corals. XVIII. Further cembranoid diterpenes from soft corals of the genus *Sarcophyton*. *Aust. J. Chem.* **1980**, *33*, 879-884. https://doi.org/10.1071/CH9800879.
2. Blackman, A.J.; Bowden, B.F.; Coll, J.C.; Frick, B.; Mahendran, M.; Mitchell, S.J. Studies of Australian soft corals. XXIX. Several new cembranoid diterpenes from *Nephthea brassica* and related diterpenes from a *Sarcophyton* species. *Aust. J. Chem.* **1982**, *35*, 1873-1880. https://doi.org/10.1071/CH9821873.
3. Xu, X.-H.; Kong, C.-H.; Lin, C.-J.; Wang, X.; Lu, J.-H. Isolation and identification of a novel cembrane-type diterpenoid from the soft coral *Sarcophyton crassocaule*. *Chem. J. Chinese U.* **2003**, *24*, 1023-1025.
4. Huang, H.-C.; Chao, C.-H.; Kuo, Y.-H.; Sheu, J.-H. Crassocolides G-M, cembranoids from the Formosan soft coral *Sarcophyton crassocaule*. *Chem. Biodivers.* **2009**, *6*, 1232-1242. https://doi.org/10.1002/cbdv.200800142.
5. Shaker, K.H.; Müller, M.; Ghani, M.A.; Dahse, H.-M.; Seifert, K. Terpenes from the soft corals *Litophyton arboreum* and *Sarcophyton ehrenbergi*. *Chem. Biodivers.* **2010**, *7*, 2007-2015. https://doi.org/10.1002/cbdv.201000016.

Table S1. X-ray crystallographic data for compound **4**

Empirical formula	$C_{20}H_{34}O_4$
Formula weight	338.47
Temperature/K	170
Crystal system	orthorhombic
Space group	$P2_12_12_1$
a/Å	9.7323(3)
b/Å	13.0447(4)
c/Å	15.4503(4)
α/°	90
β/°	90
γ/°	90
Volume/Å^3	1961.49(10)
Z	4
ρ_{calc}g/cm^3	1.146
μ/mm^{-1}	0.398
F(000)	744
Crystal size/mm^3	0.16 × 0.11 × 0.10
Radiation	Ga Kα (λ = 1.34139)
θ range for data collection/°	3.858 to 54.888
Index ranges	-11 ≤ h ≤ 11, -15 ≤ k ≤ 15, -18 ≤ l ≤ 18
Reflections collected	20764
Independent reflections	3713 [R(int) = 0.0502]
Data/restraints/parameters	3713/0/228
Goodness-of-fit on F^2	1.050
Final R indices [I>=2σ (I)]	R_1 = 0.0331, wR_2 = 0.0835
R indices [all data]	R_1 = 0.0366, wR_2 = 0.0869
Largest diff. peak and hole / e Å^{-3}	0.150 and -0.131
Flack parameter	-0.06(9)
